# Deciphering the Binding between Nupr1 and MSL1 and Their DNA-Repairing Activity

**DOI:** 10.1371/journal.pone.0078101

**Published:** 2013-10-30

**Authors:** David Aguado-Llera, Tewfik Hamidi, Rosa Doménech, David Pantoja-Uceda, Meritxell Gironella, Jorge Santoro, Adrián Velázquez-Campoy, José L. Neira, Juan L. Iovanna

**Affiliations:** 1 Instituto de Biología Molecular y Celular, Universidad Miguel Hernández, Elche (Alicante), Spain; 2 Centre de Recherche en Cancérologie de Marseille (CRCM), Institut National De La Santé Et De La Recherche Médicale (INSERM) Unit 1068, Centre National De La Recherche Scientifique (CNRS) Unit 7258, Aix-Marseille Université and Institut Paoli-Calmettes, Marseille, France; 3 Instituto de Química Física Rocasolano (IQFR), Consejo Superior de Investigaciones Científicas (CSIC), Madrid, Spain; 4 Centro de Investigación Biomédica en Red de enfermedades hepáticas y digestivas (Ciberehd), Barcelona, Spain; 5 Instituto de Biocomputación y Física de Sistemas Complejos (BIFI), Unidad Asociada IQFR-CSIC-BIFI, Universidad de Zaragoza, Zaragoza, Spain; 6 Fundación Agencia Aragonesa para la Investigación y Desarrollo (ARAID), Diputación General de Aragón, Zaragoza, Spain; 7 Departamento de Bioquímica y Biología Molecular y Celular, Universidad de Zaragoza, Zaragoza, Spain; St. Georges University of London, United Kingdom

## Abstract

The stress protein Nupr1 is a highly basic, multifunctional, intrinsically disordered protein (IDP). MSL1 is a histone acetyl transferase-associated protein, known to intervene in the dosage compensation complex (DCC). In this work, we show that both Nupr1 and MSL1 proteins were recruited and formed a complex into the nucleus in response to DNA-damage, which was essential for cell survival in reply to cisplatin damage. We studied the interaction of Nupr1 and MSL1, and their binding affinities to DNA by spectroscopic and biophysical methods. The MSL1 bound to Nupr1, with a moderate affinity (2.8 µM) in an entropically-driven process. MSL1 did not bind to non-damaged DNA, but it bound to chemically-damaged-DNA with a moderate affinity (1.2 µM) also in an entropically-driven process. The Nupr1 protein bound to chemically-damaged-DNA with a slightly larger affinity (0.4 µM), but in an enthalpically-driven process. Nupr1 showed different interacting regions in the formed complexes with Nupr1 or DNA; however, they were always disordered (“fuzzy”), as shown by NMR. These results underline a stochastic description of the functionality of the Nupr1 and its other interacting partners.

## Introduction

The mammalian MOF (male-absent-on-the-first)-MSL (male-specific-lethal) complex –also known as the “dosage compensation complex” (DCC)- has four stably-associated proteins: MOF, MSL1, MSL2 and MSL3 [1]. The dosage compensation is an essential process that equalizes the expression levels of X-chromosomal genes between males and females. The complex is a model for studying chromosome-wide transcription regulation by histone hyperacetylation [2], [3]. It is actually known that the histone acetyltransferase MOF is tightly regulated by MSL1 and MSL3 [4], [5], and it even intervenes in the outcome of autophagy [6]. In *Drosophila*, the complex also involves two functionally redundant non-coding RNAs [2]; in mammals, no associated RNA has been yet identified [1], [7], [8], and there are two MOF-MSL complexes (MOF-MSL1 and MOF-MSLv1). Both complexes are important for most of the histone 4 K16 acetylation in cells, for gene expression, cell-cycle regulation, DNA-damage repair and other regulatory processes in the nucleus [9]–[12]. In addition to the MSL complex, MOF also resides in an NSL (non-specific lethal) complex, that plays an important role in genome-wide chromatin modification and transcriptional regulation [13]–[15]. The human 614-residue-long and the 1039-residue-long *Drosophila* MSL1s are predicted to contain no globular domains (that is, they are intrinsically disordered proteins, IDPs). Both, the scaffold of the MSL complex [16] and dimerization of MSL1 coiled-coil region with the MSL2 protein are essential for male X-chromosome regulation in *Drosophila* [8], [17].

The Nupr1 gene was first described as overexpressed in acinar cells of the pancreas during the acute phase of pancreatitis [18]. The Nupr1 protein is an IDP, which binds DNA and is a substrate for protein kinase A; phosphorylation seems to increase the content of residual structure, and the phosphorylated species also binds DNA [19]. The exact function of Nupr1 is unknown, although it has been involved as a scaffold protein in transcription, and as an essential element of the defence system of the cell and in cell-cycle regulation. Furthermore, Nupr1 expression controls pancreatic cancer cell migration, invasion and adhesion, three processes required for metastasis through CDC42, a major regulator of cytoskeleton organization [20]. Also, Nupr1 seems to play a major role in pancreatic tumorigenesis since the oncogenic Kras^G12D^ expression in mice pancreas is unable to promote precancerous lesions in the absence of Nupr1 expression [21]. A complete account of the different functions of Nupr1 can be found in the literature [22], [23].

We have shown that in pancreatic cells Nupr1 regulates the DNA-repairing activity of MSL1 [24], which is one of the functions where MSL1 is involved (see above). Furthermore, surface plasmon resonance (SPR) and two-yeast-hybrid techniques suggest that there is an interaction between MSL1 and Nupr1. Although the MSL complex members have been studied during the last decade, the detailed molecular interactions of the members of the complex remain unknown, and the experimental structural features of the isolated, intact MSL1 remain elusive. The only region of MSL1 whose structure has been solved is that of the coiled-coil motif (always co-expressed with another region of MOF) [16]. Given, its DNA-repairing activity modulated by Nupr1 and its apparent importance during tumorigenesis, we have embarked in a description of the interaction between both proteins, and of both with DNA. In this work, first, we established that both Nupr1 and MSL1 proteins are recruited and form a complex into the nucleus in response to DNA-damage; we found that this complex was essential for cell survival in response to cisplatin damage. Second, we expressed, refolded and purified intact MSL1 with a TRX-tag. The protein was an oligomeric IDP, as judged by ITC and thermal denaturation experiments followed by circular dichroism (CD) and fluorescence. Next, we studied, by using different biophysical and spectroscopic techniques (namely, fluorescence, CD, NMR and ITC), the affinities of Nupr1 for MSL1 in the absence and the presence of etoposide-damaged-DNA. And finally, we described the binding site of Nupr1 towards MSL1 and DNA by triple-resonance NMR experiments. First, our results suggest that the function of MSL1 was exquisitely modulated to recognize damaged DNA. The apparent affinities of Nupr1, MSL1 or the complex MSL1 + Nupr1 for DNA were similar, and they were found in the low micromolar range (∼0.4–1.2 µM), whereas the apparent affinity of the MSL1 for Nupr1 was lower with a value of ∼2.8 µM. The binding region of Nupr1 changed from the binary complex with MSL1 to the ternary complex with MSL1 + chemically-damaged-DNA; this result suggests a fine modulation of the interactions of this protein with its different biomolecular partners.

## Materials and Methods

### Materials

Deuterium oxide and IPTG were obtained from Apollo Scientific (UK). Sodium trimethylsilyl [2,2,3,3-^2^H_4_] propionate (TSP), deuterated acetic acid and its sodium salt, calf-thymus DNA, and TFE were from Sigma (Spain). DNA restriction enzymes were from New England Biolabs (UK). Coomassie brilliant blue (R240), Triton X-100 and β-mercaptoethanol were from BioRad (Spain). Dialysis tubing, with a molecular weight cut-off of 3500 Da, was from Spectrapor (Spectrum Laboratories, Japan). Standard suppliers were used for all other chemicals. Water was deionized and purified on a Millipore system.

### Cell Culture and in vitro Stress Induction

MiaPaCa-2 cells obtained from the American Type Culture Collection were maintained in DMEM medium (Invitrogen) supplemented with 10% phosphate buffer solution (PBS) at 37°C with 5% CO_2_. To avoid any additional stress, all media were warmed at 37°C before rinsing or changing media. For DNA damage induction, 10 mM cisplatin stock solution was prepared.

### siRNA Transfection

MiaPaCa-2 cells were plated at 70% confluence in 100 mm dishes. Nupr1 and MSL1 were knocked-down using 140 ng of specific siRNAs. Scrambled siRNA targeting no known gene sequence was used as negative control. INTERFErin™ transfection reagent (POLYPLUS) was used to perform siRNA transfection according to the manufacturer’s protocol. The sequences of Nupr1 and MSL1-specific siRNAs have been previously reported [24], [25]. Statistical analysis was performed byANOVAand post hoc analysis with Studen-Newman-Keuls test. Results shown the mean ± standard deviation.

### Cell Viability and Caspase 3/7 Activity Assay

Cells were seeded in six-well plates. Cells were allowed to attach overnight, RNAi treated and finally subjected to cisplatin treatment. To determince cell viability and to detect caspase 3/7 activity, cells were seeded on ninety-six-well plates at a density of 10,000 cells/well. At the end of experiment, cell number and caspase 3/7 activity were monitored by using CellTiter-Blue® (Promega G8081) and Apo-ONE® Caspase-3/7 assay (Promega G7790), respectively. Caspase 3/7 activity was estimated as the ratio Apo-ONE to the CellTiter-Blue signals. Statistical analysis was performed byANOVAand post hoc analysis with Studen-Newman-Keuls test. Results shown the mean ± standard deviation.

### Immunofluorescence

Cells cultured on glass coverslips were treated as previously described [21], fixed, permeabilized and incubated with rabbit anti-γ-H2AX (Bethyl, A300-081A) followed by mouse Alexa Fluor anti-Rabbit IgG 488 (Invitrogen Life Technologies, Carlsbad, CA) secondary antibody. Nuclei were stained utilizing Prolong DAPI (Invitrogen Life Technologies, Carlsbad, CA). Fluorescence microscopy was performed by using a Nikon Eclipse 90i fluorescence microscope (Nikon Instruments Europe B.V). Images were captured in 16 bit TIFF format with a Nikon DS-1QM camera and visualized utilizing NIS-Elements 3.0 software. For γ-H2AX foci number counting, four independent cell fields with about 40 cells each were examined at 20x. Statistical analysis was performed byANOVAand post hoc analysis with Studen-Newman-Keuls test. Results shown the mean ± standard deviation.

### Proximity Ligation Assay (PLA)

MiaPaCa-2 cells were seeded on coverslips and transfected with 300 ng of DNA (Nupr1-Flag and MSL1-V5) using Fugene HD™ transfection reagent (Roche). At the end of the experiment, cells were washed twice in PBS, fixed, permeabilized and saturated for 45 min before immunostaining with DuoLink kit (Olink Bioscience) following the manufacturer’s protocol. Image acquisition was carried out on a Nikon Eclipse 90i fluorescence microscope. Statistical analysis was performed byANOVAand post hoc analysis with Studen-Newman-Keuls test. Results shown the mean ± standard deviation.

The qRT-PCR and western blot analysis were carried out as described previously [17]. The β-tubulin was used as a control of loading.

### Protein Expression and Purification

We carried out different attempts to obtain the human MSL1 for biophysical studies.

First, the cDNA from clone DKFZp686J17211 (Genbank accession number CR749360; Swiss-Prot number: Q68DK7) from *Homo sapiens* mRNA, encoding the 413-residue-long differentially spliced isoform was cloned the pQE30 into the BamH1/Sal1 restriction sites to obtain a His-tagged protein (the sequence of the protein is provided in Figure S1 of File S1, it contains four tryptophans and four tyrosines). The sequence of the protein was tested by DNA sequencing. Expression attempts by using BL21(DE3), BL21(DE3)pLys, C41, C43 and Rosetta strains were carried out at 15, 25 and 37°C, with several IPTG final concentrations ranging from 0.2 to 1 mM. In all cases, the expression was unsuccessful. Therefore, we were not able to obtain pure MSL1, with a short tag.

Next, the protein was cloned, by using the services of GenScript (New Jersey), into three vectors: (i) pET-32a (into the *Kpn*I/*Xho*I restriction sites) to obtain a His-tagged TRX-MSL1 fusion protein with a 6× His-tag incorporated with a (thioredoxin) TRX-tag at the N terminus of the protein; (ii) pGS21a (into the *Kpn*I/*Xho*I restriction sites), which contains a GST (glutathione S- transferase) at the N terminus of the protein; and, (iii) pCold TF (into the *Nde*I/*Xba*I restriction sites), with the TF (trigger factor) at the N-terminus of MSL1, which helps to solubilise proteins. In the three plasmids, we tried expression in ArcticExpress (DE3), BL21(DE3), BL21(DE3)pLys, C41, C43 and Rosetta strains at 15, 25 and 37°C, with IPTG final concentrations of 0.2, 0.5 and 1 mM. Expression was obtained for the peET-32a in all the strains at any IPTG concentrations and temperatures, but in all cases the proteins was expressed as inclusion bodies. Expression with pGS21a and pColdTF was obtained with the ArcticExpress (DE3), C41 and Rosetta strains at any temperature and IPTG concentrations; however, the proteins were also expressed as inclusion bodies, and the yields, as judged by visual inspection in SDS-PAGE gels were lower than those for the pET-32a plasmid. Therefore, since: (i)we had to extract the protein, in all cases, from inclusion bodies; (ii) the length of the TRX-tag was smaller than those of the GST and TF; and (iii) we had a larger experience in refolding TRX-tagged proteins [26], we decided to work with the pET-32a plasmid and elaborate a refolding protocol.

We transformed C41 *E. coli* cells [27] with the pET-32a. Cells were incubated overnight at 37°C, in the presence of ampicillin (50 µg/ml). This culture was then inoculated in four two-litre flasks containing LB media supplemented with 50 µg/ml ampicillin. The culture was incubated at 37°C until the OD_600_ reached a value of 0.6–0.9. Protein was overexpressed by the addition of IPTG at a final concentration of 1 mM, and overnight incubation at 37°C. We also tried with this strain a shorter growing time after induction (three and six hours), but under these conditions, inclusion body formation was observed. The resultant culture was centrifuged at 4°C during 15 min at 6000×g in a Beckman Coulter J2-HS centrifuge (Germany). The cell pellets were frozen at −80°C until processing. The supernatant was discarded. Cell pellets from 4 l of LB media were resuspended in 50 ml of lysis buffer (500 mM NaCl, 5 mM imidazole, 20 mM Tris (pH 8.0), 0.1% Triton X-100 and 1 mM β-mercaptoethanol) and one tablet of complete-EDTA-free protease inhibitor (Roche, Germany). Sonication was carried out by bursting the suspension in ice 10 times, during 45 s, with interleaved periods of 15 s in ice, at the maximum power of the sonicator (Model 102-C, Branson, USA). The resultant solution was centrifuged for 50 min at 27000×g. Since the supernatant did not contain any evidence of MSL1, it was discarded and the cellular pellet was resuspended in 50 ml of lysis buffer plus 7 M GdmCl, with a tablet of complete-EDTA-free protease inhibitor. The resuspended solution was sonicated and centrifuged again, by using the same conditions described above. The supernatant was dialyzed against buffer A (50 mM acetate buffer (pH 4.5) with 6 M NaCl) to remove GdmCl. The buffer A was changed three times, and then substituted by buffer B (50 mM acetate buffer (pH 4.5) with 3 M NaCl). The buffer B was changed three times and, then, substituted by buffer C (50 mM acetate buffer (pH 4.5) with 1 M NaCl). The buffer C was changed three times and then substituted by 50 mM acetate buffer (pH 4.5). Finally, acetate buffer was changed three times and the resulting sample was centrifuged at 27000×g for 40 minutes to eliminate the precipitate from the dialysis tubing. In this step-by-step dialysis-process protein precipitation was minimized. Attempts to dialyze the TRX-tagged-MSL1 at pH >4.5 caused protein precipitation, probably because of scrambling and disulphide bridge formation due to the cysteines in its sequence (8 Cys). The resulting supernatant contained TRX-tagged-MSL1, reaching a purity larger than 80% as judged by 12% SDS-PAGE. The protein concentration was determined by measuring the absorbance at 280 nm with the extinction coefficients as determined from amino acid sequence [28]. Absorbance measurements were carried out in a Shimadzu UV-1601 ultraviolet spectrophotometer using a 1-cm-path-length cell (Hellma). The final protein yield after the purification protocol was 2.5 mg per litre of culture.

Nupr1 was produced and purified in LB media as described [19]. For the production of ^15^N- or ^15^N, ^13^C- double- labelled samples the cells were grown in M9 minimal media, supplemented with vitamins, and purified as the protein grown in LB media [19].

### Production of Chemically-damaged DNA

DNA from calf-thymus was incubated in the presence of etoposide (100 µM in (2,2,2-trifluoroethanol) TFE, Calbiochem, USA), to a final DNA concentration of 1 mg/ml. Samples were incubated at 4°C overnight to cause double- and single-strand breaks in DNA. We used TFE to dissolve etoposide, instead of the most commonly used DMSO, to avoid interferences in the acquisition of far-UV CD spectra. We carried out controls up to 10% (vol/vol) of TFE to be sure that the residual structure of MSL1, if any, was not altered upon TFE addition. The structure of Nupr1 is not altered upon TFE addition, as previously shown [19].

It is important to note that we do not have a proper assessment of the DNA damaged inflicted by etoposide, and that is generally admitted that it causes single- and double- strand breaks. From the NMR experiments (see below), it is clear that the size of the DNA-etoposide-treated is smaller than that of the original DNA.

### DNA Binding Experiments

For the binding experiments with DNA (either damaged or non-damaged), far-UV CD and fluorescence spectra were acquired at 20 µM of each protein (MSL1 and/or Nupr1) and an equal amount (in mg) of damaged- or non-damaged- DNA, at pH 4.5 (50 mM acetate buffer). Far-UV CD and fluorescence spectra were acquired for each isolated protein (at the same concentration), and, in addition, for MSL1 + Nupr1, MSL1 + DNA, Nupr1 + DNA, and MSL1 + Nupr1 + DNA complexes at 25°C. For the binding experiments with DNA, we used calf-thymus DNA, because: (i) its binding to Nupr1 has been previously described [19]; and, (ii) the DNA-sequence specifically recognized by Nupr1 is not known. We also carried out measurements at 20 µM of TRX-tag to rule out the possibility of DNA binding, by using fluorescence or CD; no variation of the TRX-tag spectral features in the presence of DNA was observed by any technique when compared to those in its absence. The isolated TRX-tag was obtained as described [26].

### Far-UV CD Experiments

Far-UV CD spectra were acquired in a Jasco J810 spectropolarimeter (Jasco, Japan) with a thermostated cell holder and interfaced with a Neslab RTE-111 water bath, using a 0.1-cm-path-length cell, a scanning speed of 50 nm/min and a response time of 4 s. The instrument was periodically calibrated with (+) 10-camphorsulphonic acid. The spectra reported are the average of six scans at 25°C. We have preferred to work with the raw ellipticity, since different complexes were obtained. Spectra were corrected by subtracting the corresponding blank.

Thermal denaturations were carried out for each of the isolated proteins and the corresponding complexes, by applying a constant heating rate of 60°C/h; the ellipticity was monitored at 222 nm from 25 to 95°C. The response time was 8 s, the bandwidth was 1 nm and the data were acquired every 0.2°C in 0.1-cm-path-length cells. Every thermal denaturation was repeated three times with new samples.

### Fluorescence Experiments

Fluorescence spectra were acquired in a Varian Cary Eclipse spectrofluorimeter (Agilent, USA) interfaced with a Peltier unit. The excitation wavelengths were either 278 or 295 nm; the emission fluorescence was collected between 300–400 nm. The excitation and emission slits were 5 nm and the data pitch interval was 1 nm at 25°C. Spectra were corrected by subtracting the corresponding blank.

Thermal denaturations were carried out by excitation at 278 and 295 nm, and collected at 315, 330 and 350 nm. Slit widths were 5 nm for excitation and emission lights. Thermal scans were acquired every 0.2°C with a heating rate of 60°C/h, and averaged every 0.1 s. Every thermal denaturation was repeated three times with new samples.

### Agarose Gels

Fresh samples were dissolved in standard DNA electrophoresis loading buffer. Samples were loaded in 2% agarose gels with 1∶10000 of GelRed fluorescent dye (Biotium, Hayward, CA, USA) and they were run in TAE buffer. UV trans-illumination was used to visualize DNA migration. Gel images were acquired in a TDI GelPrinter Plus system connected to a PC computer.

### ITC (Isothermal Titration Calorimetry) Experiments

ITC titrations were carried out by using an isothermal titration calorimeter Auto-ITC200 (MicroCal, GE Healthcare). Measurements were carried out at 25°C in acetate buffer, 10 mM (pH 4.5). Samples were degassed for 10 min at room temperature with gentle stirring before being loaded into the calorimeter. Typically, 40 µM MSL1 solution was injected into 4 µM Nupr1 solution, 0.03 mg/ml etoposide-damaged-DNA, and both ligands together (at the same concentration used when experiments were carried out with the isolated reagents). In addition, the interaction involving Nupr1 and etoposide-damaged-DNA was also studied; in that case, the sample cell was loaded with 0.03 mg/ml etoposide-damaged-DNA solution and the syringe with 40 µM Nupr1 solution. In all the assays, a total of 19 injections of 2 µl were made sequentially to the sample cell after a 150-s spacing to ensure that the thermal power returned to the baseline before the next injection. The amount of thermal power required to maintain the sample cell at a constant temperature after each injection was monitored as a function of time. It is important to indicate that all the ITC measurements were carried out with etoposide-damaged-DNA, since MSL1, as concluded from the spectroscopic measurements (see below), did not bind to non-damaged-DNA.

The individual dilution heats for Nupr1 and MSL1 were determined, under the same experimental conditions, by carrying out identical injections of the corresponding protein into the sample cell, which contained buffer only. The Nupr1 protein did not show evidence of dilution heat, thus, suggesting that the protein remained monomeric under these conditions. On the other hand, MSL1 did show evidence of dilution effects, and we concluded that the protein was an oligomer. The heat of dilution for MSL1 was employed as a reference data for analyzing all assays in which MSL1 was injected into either DNA and/or Nupr1.

The isotherms (normalized differential heat upon binding *versus* the molar ratio of the reagents in the cell) were fit to a single-site binding model. The experiments allowed determining simultaneously the binding affinity, the binding enthalpy and the stoichiometry (the parameter c, Wiseman constant, defined as [macromolecule in cell]/*K*_D_ was significantly larger than 1 in all cases). Data were analyzed with software developed in our laboratories, implemented in Origin 7.0 (OriginLab). The molecular weight of DNA was assumed to be 10^4^ kDa to estimate the apparent *K*_D_s.

### NMR Experiments

The NMR data were acquired at 25°C on a Bruker Avance DRX-500 spectrometer equipped with a triple-resonance probe and z-gradients, and on a Bruker AV 800 spectrometer equipped with an inverse triple-resonance cryoprobe and z-gradients. 2D ^1^H-^15^N HSQC (heteronuclear single-quantum coherence) experiments [29] for: (i) ^15^N-labelled Nupr1 (100 µM); (ii) TRX-tag (100 µM) and ^15^N-labelled Nupr1 (100 µM); (iii) ^15^N-labelled Nupr1 (100 µM) and calf-thymus (non-damaged) DNA (0.5 mg/ml to minimize viscosity); and (iv) ^15^N-labelled Nupr1 and etoposide-damaged-DNA (0.5 mg/ml to minimize viscosity) were acquired in the Avance DRX-500 spectrometer.

Spectra were acquired in the phase sensitive mode. Frequency discrimination in the indirect dimensions was achieved by using the echo/antiecho-TPPI method. The spectra were acquired with 1 K complex points in the ^1^H dimension, 128 complex points in the ^15^N dimension, and 200 scans. The carrier of the ^1^H dimension was set at the water frequency, and that of ^15^N at 120 ppm. The spectral widths used were 10 and 35 ppm in the ^1^H and ^15^N dimensions, respectively. Water was suppressed with the WATERGATE sequence [30]. Data were zero-filled to double the number of original points in both dimensions, apodized with shifted squared sine-bell functions in the two dimensions and Fourier transformed with the program TopSpin 1.3. Overlay of 2D ^1^H-^15^N HSQC spectrum of Nupr1 + TRX-tag with that of isolated Nupr1 did not show any differences, suggesting lack of binding (data not shown). Moreover, the ^15^N-HSQC spectrum of non-chemically damaged DNA with ^15^N-labelled Nupr1 did not show any signal (see below), suggesting that both molecules were bound. On the other hand, the ^15^N-HSQC spectrum with etoposide-damaged-DNA showed disappearance of some signals and movement of others (see below). The absence of signals in the spectrum with non-damaged DNA was likely due to the significant increase in molecular tumbling time through the formation of a large molecular weight complex. In turn, the presence of signals in the spectrum with chemically-damaged-DNA suggests that the size of the DNA fragments is smaller. It could be argued that smaller molecular complexes (and therefore better HSQC spectra) could be obtained with designed, shorter DNA fragments; however, we do not know the exact DNA sequence recognized by Nupr1 and we started working with calf-thymus DNA in our first studies with Nupr1 [19].

For the sequence-specific assignment of Nupr1, 1.7 mM of an uniformly ^13^C- and ^15^N-labelled Nupr1 in 10 mM acetic/acetate buffer, pH 4.5, and 9∶1 H_2_O:D_2_O was used. The assignment was obtained by standard triple resonance techniques [31] using 2D ^1^H-^15^N HSQC and 3D HNCO, HN(CA)CO, intra-HNCA, HN(CO)CA, intra-HNCACB and CBCA(CO)NH spectra acquired in the AV 800 spectrometer. The spectra were acquired with 1 K complex points in the ^1^H dimension, 24 complex points in the ^15^N dimension and 45 or 50 complex points in the ^13^C dimension, and 4 or 8 scans. The resulting matrix was zero-filled to double the number of original points in all dimensions and shifted squared sine-bell apodization functions were applied in all dimensions prior to Fourier transformation. The programs NMRPipe [32] and NMRView [33] were used for spectral processing and data analysis, respectively. In addition, 2D experiments that provide amino acid type identification of the NH correlation signals of the ^1^H-^15^N HSQC spectrum were used to facilitate the assignment [34], [35]. A list of assignments of the backbone nuclei of Nupr1 is given in Table S1 in File S1. Assignments were referenced in the three dimensions as described [36]; the BMRB (Biomagnetic Resonance Bank) accession number for the assignment of Nupr1 is 19364.

To study the binding of Nupr1, 2D ^1^H-^15^N HSQC experiments of the isolated protein and of the complexes were also acquired in the AV 800 spectrometer. For the backbone assignment, similar methodology to that of isolated Nupr1 was used. The 95% and 70% of the peaks of 2D ^1^H-^15^N HSQC spectrum could be assigned for Nupr1 + MSL1 complex and Nupr1 + MSL1 + etoposide-damaged-DNA complex, respectively. The 2D spectra were acquired with 1024 complex data points in the ^1^H dimension, 128 complex points in the ^15^N dimension, a relaxation delay of 1 s and 8 transients *per* fid. The total acquisition time of each 2D spectrum was 1 h 25 min. Before Fourier transform, time-domain data were zero-filled to a 512×2048 hypercomplex data matrix and apodized with shifted squared sine-bell functions in the two dimensions. The programs NMRPipe [32] and NMRView [33] were used for spectral processing and data analysis, respectively. For the experiment containing only Nupr1, protein concentration was 100 µM; for the binding experiments to MSL1, the concentration of every protein was 90 µM; and in the experiments with MSL1 and damaged DNA, the final concentration of each protein was 90 µM, and that of nucleic acid was 0.5 mg/ml (to minimize viscosity of the NMR sample).

To analyse the differences between the 2D ^1^H-^15^N HSQC spectra of isolated Nupr1 and of the complexes, the chemical shift perturbation (CSP) was used. CSP was calculated as: 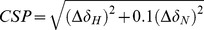, where 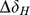 is the difference in chemical shift of the amide protons of isolated Nupr1 and that of Nupr1 in the complex; and 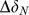 is the difference in chemical shift of ^15^N resonances of isolated Nupr1 and that of Nupr1 in the complex.

## Results

We followed a two-part strategy. We first measured the Nupr1 and MSL1 interaction with DNA in the cells, and how that interaction modulated the DNA-repairing activity of MSL1. And second, we measured quantitatively the strength of that interaction, and the Nupr1 interacting region *in vitro* by using spectroscopic and biophysical techniques.

### Nupr1 and MSL1 Impaired Cisplatin-induced DNA Damage

**Figure 1:**
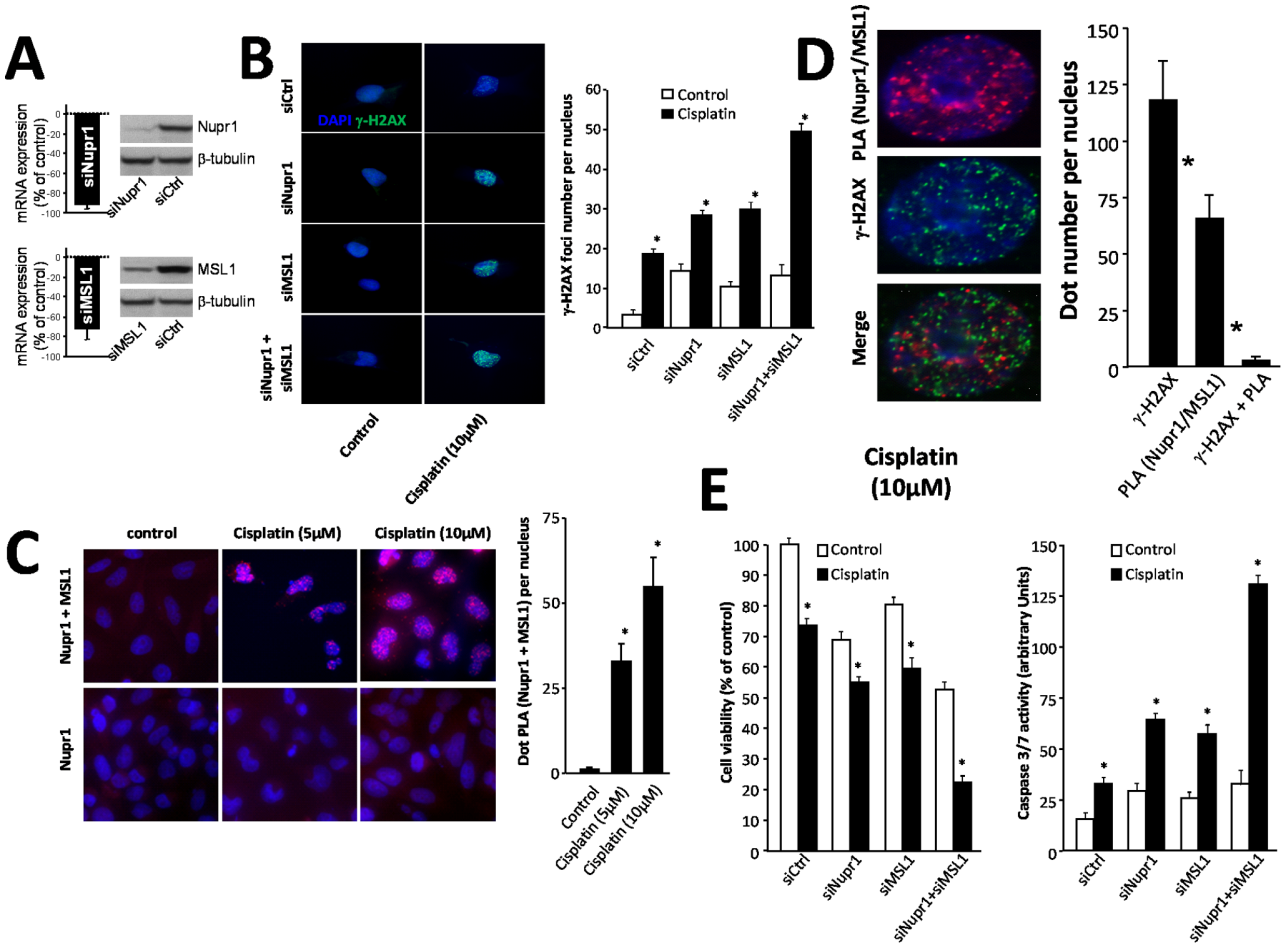
Cell survival and caspase 3/7 activity in response to cisplatin treatment and Nupr1 and MSL1 interaction. (A) MiaPaCa-2 cells were transfected with siNupr1 or siMSL1 and cultured in conventional media for an additional 24-h period; Nupr1 and MSL1 mRNA expression was measured by qRT-PCR, and proteins levels by western blot analysis. β-tubulin was used as a control of loading. (B) MiaPaCa-2 cells were plated on coverslips and transfected with siNupr1 or siMSL1, alone or together, and 24 h later treated with cisplatin (10 µM) for a 24 h-period. γ-H2AX staining was performed by immunofluorescence. The 40× magnification was used to count the number of γ-H2AX dots. Data are the means of 10 field counting with not less than 100 nucleus counted (* p≤0.05). (C) Proximity Ligation Assay (PLA) of Nupr1 and MSL1. Cells were plated on coverslips and transfected with pcDNA3-Nupr1-Flag and pcDNA4-MSL1-V5 constructs. The day after the experiment, cells were treated with cisplatin (5 µM or 10 µM) to induce DNA damage and 24 h later the PLA was performed as described in Material and methods section. Red dots represent Nupr1/MSL1 interaction. DNA transfection with only pcDNA3-Nupr1-Flag construct was used as a negative control. (D) Nupr1 and MSL1 do not interact into the DNA damage sites. PLA was reproduced as in (B) and followed by γ-H2AX staining. A 20 fields of 40× magnification were used to count the number of γ-H2AX green dots, the number of PLA red dots and the number of co-localizing green and red dots. Data are the means of 20 field counting with not less than 100 nucleus counted (* p≤0.05). (E) MiaPaCa-2 cells were plated in six-well plates and treated with cisplatin (30 µM) for 24 h. Cell survival rate was estimated using a cell counter as described in the Material and Methods section. Data are expressed as percentage of the control. Cells were plated in ninety-six-well plates treated with cisplatin as described above and caspase 3/7; we assayed the caspase acativity by using the Apo-ONE® homogenous caspase 3/7 assay. The normalization value was performed by using CellTiter-Blue® viability assay according to manufacturer’s instructions (* p≤0.05).

Previous studies have shown that Nupr1 regulates the MSL1-dependent DNA repair activity after irradiation-induced DNA-damage using a clonogenic survival assay [24]. To test whether Nupr1 and MSL1 were involved in DNA repair, MiaPaCa-2 cells were treated with cisplatin, after silencing Nupr1 and MSL1 expression. In all cases, control qRT-PCR experiments were carried out to test for mRNA expression levels (Fig. 1 A). The DNA-damage was measured by γ-H2AX immuno-staining (Fig. 1 B, left side). Nupr1 and MSL1 silencing (alone or together) slightly increased DNA damage in untreated cells. However, in cisplatin-treated cells, the γ-H2AX foci number *per* nucleus increased from 19.1±2.2 in siCtrl-transfected cells to 28.1±2.3, 29.6±3.8 and 48.6±4.1 in siNupr1-, siMSL1- and in siNupr1-siMSL1-treated cells, respectively (Fig. 1 B, right side). These data suggest that both Nupr1 and MSL1 proteins were required to protect cells from cisplatin-induced DNA damage.

### DNA-damage Triggered Nupr1 and MSL1 Interaction

Then, we monitored Nupr1 and MSL1 interaction in MiaPaCa-2 cells subjected to DNA-damage using the PLA (Fig. 1 C, left side); indeed, PLA allows direct visualization of a given interaction directly into the cell. Interestingly, we found that treatment of MiaPaCa-2 cells with two doses of cisplatin triggered Nupr1 and MSL1 interaction in a dose-dependent manner (32±4.8 and 57±6.2 dots PLA *per* nucleus with 5 and 10 µM cisplatin respectively (Fig. 1 C, right side)). Furthermore, the Nupr1/MSL1 interaction was located almost exclusively into the nucleus. These findings suggest that Nupr1 and MSL1 proteins could be recruited near, or into, the DNA-damaged site and could be implicated in DNA-repair activity. To test this hypothesis, we combined PLA assay with γ-H2AX staining in MiaPaCa-2 cells treated with cisplatin. The Nupr1 + MSL1 complex (red) did not co-localize with γ-H2AX staining (green) (Fig. 1 D, left side); in fact, we found 119±18.1 γ-H2AX foci and 53 PLA dots, but only 4±0.5 co-localizations (Fig. 1 D, right side). Altogether, these data demonstrate that Nupr1 interacted with MSL1 in response to DNA-damage, but this interaction did not occur on the DNA-damaged sites.

### Nupr1 and MSL1 Protected Pancreatic Cancer Cells against DNA-damage-induced Cell Death

As described above, Nupr1 and MSL1 were involved against DNA-damage. Nupr1 is a stress-related protein involved in cell survival in response to several stresses [23]. We used specific siRNAs to test whether Nupr1 and MSL1 partners are involved in pancreatic cancer cell survival after DNA damage induced by cisplatin treatments. Cell survival experiments show that Nupr1 or MSL1 single silencing slightly decreased cell survival after cisplatin treatment from 73±2.3 in siCtrl transfected cells to 54.1±3.1 and 57.9±4.3 in siNupr1- and siMSL1-transfected cells, respectively. Most importantly, we found that knocking-down simultaneously Nupr1 and MSL1 expression dramatically decreased cell survival upon cisplatin treatment (21.1±2.0) (Fig. 1 E, left side). Additionally, caspase 3/7 activity shows that the decrease of cell survival rate was related to an increase of caspase activity. We found 33±3 arbitrary units in siCtrl-transfected cells treated with cisplatin and 64±2, 58±4 and 129±8 units, in siNupr1-, siMSL1- and siNupr1-siMSL1-transfected cells, respectively (Fig. 1 E, right side). These data suggest that upon DNA damage, Nupr1 and MSL1 were strongly involved in pancreatic cancer cell protection through the inhibition of apoptotic-induced cell death.

### MSL1 showed Features of IDPs

We studied the conformational propensities of isolated MSL1 to see whether upon addition of other biomolecules these features were changed. Besides, there are no studies of such kind with the whole, intact MSL1.

**Figure 2:**
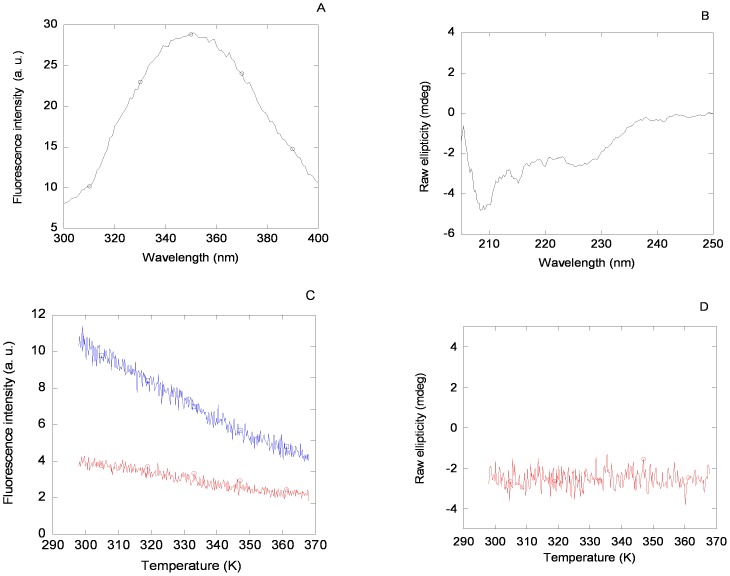
Fluorescence and CD spectra of MSL1. Fluorescence (A) and far-UV CD (B) spectra for MSL1 in 50 mM acetic/acetate buffer (pH 4.5). Conditions for both experiments were 20 µM of protein and 25°C. Thermal denaturation of MSL1 followed by fluorescence (C) and far-UV CD (D). Fluorescence lines represent the thermal denaturations of MSL1 at an emission wavelength of 315 nm, either at an excitation wavelength of 278 nm (blue line) or 295 nm (red line). Similar results were obtained when emission wavelength was monitored at 330 nm or 350 nm. Spectra were acquired in either 1 cm- (fluorescence) or 0.1 cm- (far-UV CD) path-length cells.

The fluorescence emission spectrum of MSL1 was dominated by its four tryptophan residues and presented a maximum at 350 nm, when excited either at 280 or 295 nm (Fig. 2 A); however, it must be taken into account that the MSL1 has an attached TRX-tag, which also contains solvent-exposed tryptophan residues [37]. Thermal transitions followed by fluorescence did not show any sigmoidal behaviour (Fig. 2 C).

The far-UV CD spectrum shows a small percentage of α-helix and β-sheet, which could be due to the presence of the TRX-tag (Fig. 2 B). The shape and intensity of this spectrum was protein-concentration dependent, as it would correspond to a self-associating protein (in agreement with ITC experiments, see Materials and Methods). No sigmoidal transition was observed in thermal denaturations followed by far-UV CD at pH 4.5 (Fig. 2 D).

Therefore, the thermal denaturations followed by CD and fluorescence of the isolated MSL1 show that the secondary and tertiary structures of the protein, if any, were not stable.

### MSL1 Bound to Nupr1

**Figure 3:**
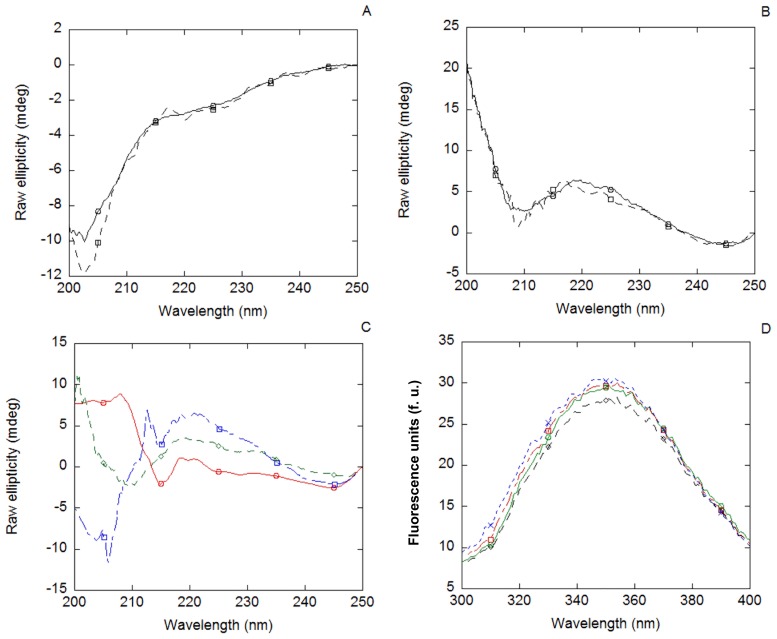
Binding of MSL1 to Nupr1 non-damaged-DNA followed by spectroscopic techniques. (A) Far-UV CD spectrum of the MSL1 + Nupr1 complex (continuous line) and the addition spectrum resulting of the sum of the spectra of each isolated biomolecule (dashed line). (B) Far-UV CD spectrum of MSL1 + non-damaged-DNA complex (continuous lines) and the addition spectrum resulting of the sum of the spectra of each biomolecule (dashed line). (C) Far-UV CD spectrum of the MSL1 + non-damaged-DNA + Nupr1 complex (red line), the addition spectrum resulting of the sum of the spectra of each isolated biomolecule (blue line) and the addition spectrum resulting of the sum of the spectra of isolated MSL1 with that of the Nupr1 + non-damaged-DNA complex (green line). Differences between the ternary complex spectrum and both sum spectra suggest that MSL1 binds to the non-damaged-DNA + Nupr1 complex. (D) Fluorescence spectrum of MSL1 (red line) compared with those of MSL1 + Nupr1 complex (blue line), MSL1 + non-damaged-DNA complex (green line) and MSL1 + Nupr1 + non-damaged-DNA complex (black line).

The fluorescence spectrum of the Nupr1 + MSL1 complex was similar to that obtained from the addition of the spectra of both isolated proteins obtained separately. That is, if they interact, binding did not alter the environment around the tryptophan residues of MSL1. However, when analyzing the MSL1 + Nupr1 complex by far-UV CD (Fig. 3 A), subtle differences could be observed in the region between 200 and 210 nm, indicating slight changes in the secondary structures of one, if not both, species, upon binding (see below).

We also recorded a 2D ^1^H-^15^N HSQC spectrum with an equimolar amount of MSL1 and ^15^N-labelled Nupr1 (Figure S2 of File S1). Several peaks of this spectrum became broad and even some of them disappeared completely. Among the peaks that disappeared were the polypeptide patch Lys66-Thr69 and residues Tyr31, Ala34-His35, Leu71, Lys77 and Arg79, which may be involved in direct binding to MSL1. There were also some residues of Nupr1 whose chemical shifts changed in the presence of MSL1. The residues showing the largest CSPs (larger than the standard deviation) were: Gly1, Thr9-Gln14, Gly17-Asp22, Leu25-Asp29 and Lys70-Asn73. The large CSP of these amino acids could be due to a different chemical environment because of conformational changes upon binding; alternatively, the environment around those residues could be altered upon binding because of a loss of flexibility or stabilization of one of the various conformers populated in the free, disordered state of Nupr1.

The ITC experiments further supported binding between both species. The *K*_D_ of the MSL1 + Nupr1 complex was 2.8±0.7 µM; the reaction was entropically-driven, but the enthalpy was unfavorable (Table 1). Therefore, the binding was characterized by a moderate affinity and, probably, driven by desolvation of protein binding interfaces.

**Table 1:** Interaction parameters of MSL1, Nupr1 and complexes at 25°C, in buffer acetate (10 mM, pH 4.5).

Structure	*K*_D_ (µM)	Δ*H* (kcal mol^−1^)
Nupr1 + etoposide-damaged-DNA	0.4±0.1	−23±2
MSL1 + etoposide-damaged-DNA	1.2±0.2	29±3
MSL1 + Nupr1	2.8±0.7	39±5
MSL1 + (Nupr1 + etoposide-damaged-DNA)^a^	0.8±0.1	28±2

a Binding of MSL1 to the previously preformed complex between Nupr1 and etoposide-damaged-DNA.

We wondered whether upon binding both proteins became ordered, as it happens in other IDPs [38]. The absence of large changes in the observed resonances of Nupr1 (Figure S1 of File S1) suggested that Nupr1 remained mostly disordered upon binding to MSL1. This suggestion was further confirmed by the lack of sigmoidal curves in the thermal denaturations of the MSL1 + Nupr1 complex followed by far-UV CD (data not shown).

### MSL1 did not Bind to Isolated Non-damaged-DNA, but it did Bind to the Non-damaged-DNA + Nupr1 Complex

The far-UV CD spectrum, obtained from the sum of the spectra of isolated MSL1 and non-damaged-DNA, was very similar to that of the corresponding complex (MSL1 + non-damaged-DNA), suggesting that no changes in the secondary structure of MSL1 occurred in the presence of non-damaged-DNA (Fig. 3 B). Thermal denaturations of the complex, followed by far-UV CD, did not show any sigmoidal behaviour. In addition, the fluorescence spectrum of MSL1 did not show a variation upon addition of non-damaged DNA (data not shown).

On the other hand, when MSL1 was incubated with the previously formed complex Nupr1 + non-damaged-DNA, the far-UV CD spectrum of the ternary-complex differs clearly from that obtained by adding those of the three isolated molecules (Fig. 3 C). These data indicate that, although Nupr1 binds to non-damaged-DNA (as previously tested [19]), the spectrum of the ternary-complex might monitor the conformational changes occurring in MSL1 upon binding to the preformed complex. However, from these experiments it was not possible to discriminate whether MSL1 bound to the complexed Nupr1 or alternatively to DNA. Thermal denaturations of the ternary complex, followed by far-UV CD, did not show any sigmoidal behaviour.

In using fluorescence to describe the tertiary complex, it is important to note that the spectrum of TRX-tagged MSL1 dominates over the spectrum of Nupr1 (whose sequence only contains two tyrosine residues [19]). The fluorescence spectrum of the ternary complex (MSL1 + non-damaged-DNA + Nupr1) had a reduction in intensity when compared to the spectrum of isolated MSL1; these results suggest that in the binding process, some of the fluorescent residues of MSL1 might be involved (Fig. 3 D). However, thermal denaturations of the ternary complex, followed by fluorescence, did not show any sigmoidal behaviour (data not shown). As TRX-tag was not cleaved from MSL1, we tested the binding of isolated TRX-tag to Nupr1 and non-damaged-DNA; no changes were observed in the far-UV CD or fluorescence spectra of TRX-tag upon addition of Nupr1 or DNA. Therefore, we conclude that spectroscopic changes were due to MSL1.

**Figure 4:**
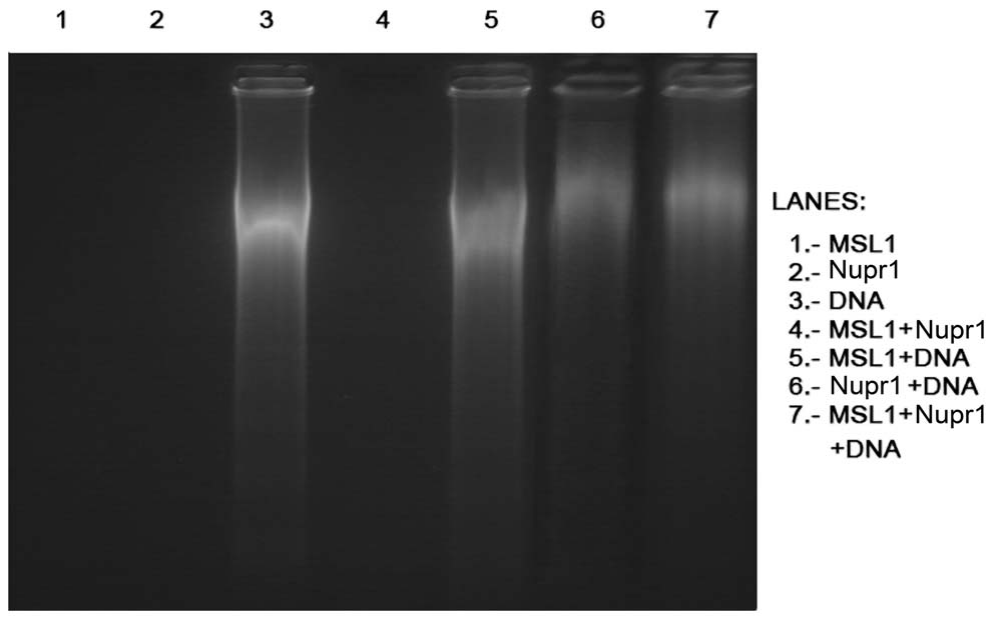
The 2% agarose gel of the binding experiments. Samples were loaded on a 2% agarose gel in TAE (1x) buffer. Lanes containing Nupr1 + non-damaged-DNA (lane 6) and MSL1 + Nupr1 + non-damaged-DNA (lane 7) were clearly delayed from DNA control lane (lane 3), indicating that there is binding between the molecular partners. No difference was observed in MSL1 + non-damaged-DNA complex (lane 5). Lanes loaded with only protein (lanes: 1 – isolated MSL1; 2 – isolated Nupr1; and 4 – MSL1 + Nupr1 complex) showed no signal.

The 2% agarose gels also showed a change in the migration pattern of the ternary complex when compared to other complexes. The gels presented a similar migration distance for isolated non-damaged-DNA and MSL1 + non-damaged-DNA complex suggesting a lack of binding of MSL1 to non-damaged-DNA. However, Nupr1 + non-damaged-DNA, and MSL1 + Nupr1 + non-damaged-DNA complexes had a reduction in the migration distance (Fig. 4). This could be due to the binding of the complex to non-damaged-DNA, causing an increase in the molecular weight of the whole complex and, leading to a shorter migration distance.

### MSL1 Bound to the Etoposide-damaged-DNA and the Etoposide-damaged-DNA + Nupr1 Complex

**Figure 5:**
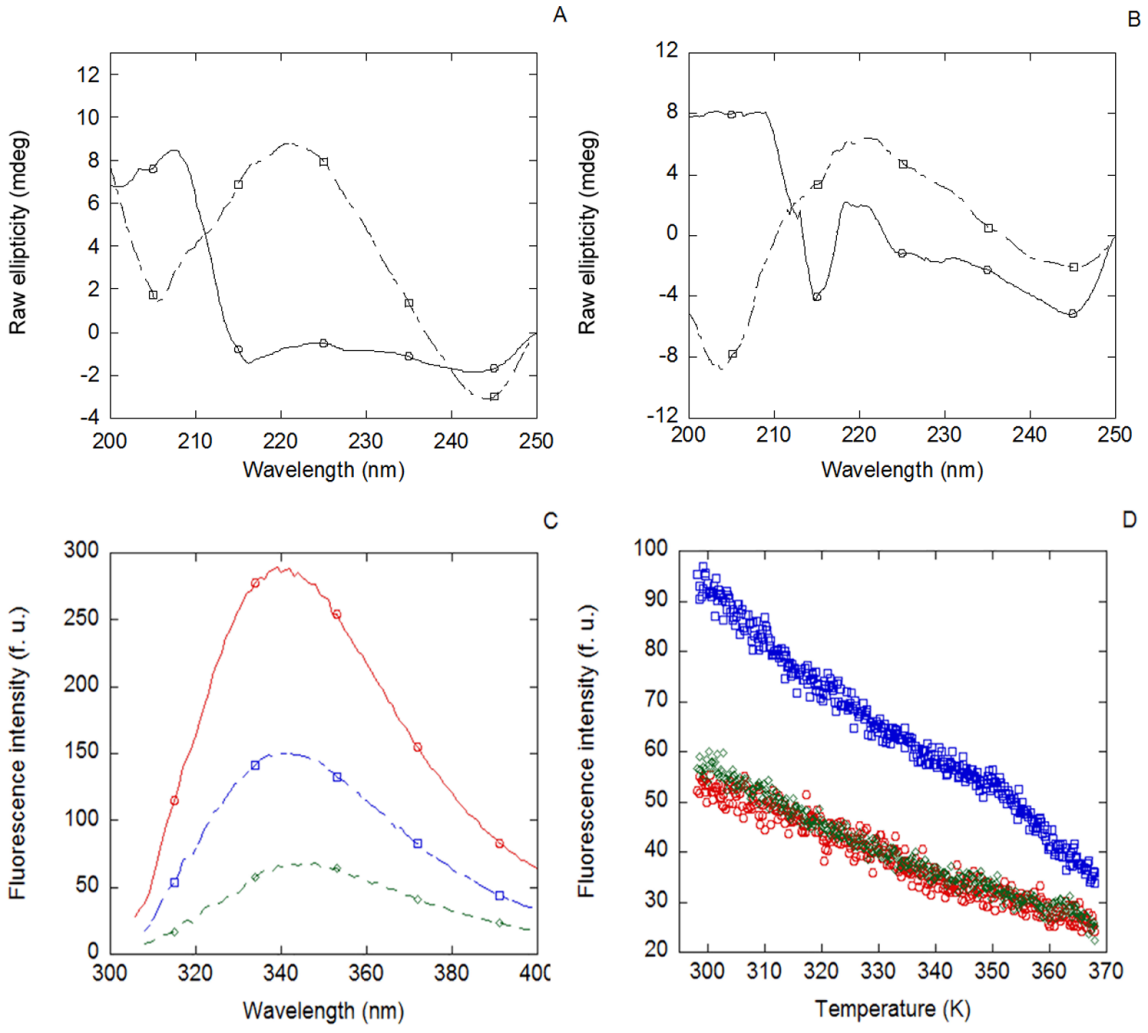
Binding of MSL1 to Nupr1 and/or etoposide-damaged-DNA followed by spectroscopic techniques. (A) Far-UV CD spectrum of MSL1 + etoposide-damaged-DNA complex (continuous line) and the corresponding addition spectra resulting of the sum of the spectra of each biomolecule (dashed lines). (B) Far-UV CD spectrum of MSL1 + Nupr1 + etoposide-damaged-DNA complex (continuous line) and the corresponding addition spectrum resulting of the sum of the spectra of each biomolecule (dashed lines). (C) Fluorescence spectra of MSL1 (red line) and the complexes of MSL1 + etoposide-damaged-DNA (blue line) and MSL1 + Nupr1 + etoposide-damaged-DNA (green line). (D) Thermal denaturations at 315 nm followed by fluorescence at an excitation wavelength of 295 nm of MSL1 (red circles), MSL1 + etoposide-damaged-DNA (blue squares) and MSL1 + Nupr1 + etoposide-damaged-DNA (green diamonds). No sigmoidal behaviour was found on any of the complexes. Similar results were obtained by monitoring the fluorescence at 330 and 350 nm.

Etoposide-damaged-DNA was also incubated in the presence of MSL1 and Nupr1, either together or separately. MSL1 bound to etoposide-damaged DNA, since the far-UV spectrum of the complex was different from that obtained by adding the spectra of each biomolecule (Fig. 5 A). Furthermore, MSL1 bound to the previously formed Nupr1 + etoposide-damaged-DNA complex (Fig. 5 B). No sigmoidal behaviour was observed in the thermal denaturations of the complexes, followed by far-UV CD.

The evaluation of the etoposide-damaged-DNA binding of MSL1, followed by fluorescence, was impaired at 280 nm because of the high emission fluorescence showed by the etoposide. However, by excitation at 295 nm, the spectra of the complexes showed a decrease in the fluorescence intensity when compared with that of the isolated MSL1 (Fig. 5 C). These results suggest that the environment of any of the tryptophans of MSL1 was modified upon addition of Nupr1 and/or etoposide-damaged-DNA. No sigmoidal behaviour was observed in the thermal denaturations, followed by fluorescence, in any of the complexes (Fig. 5 D).

**Figure 6:**
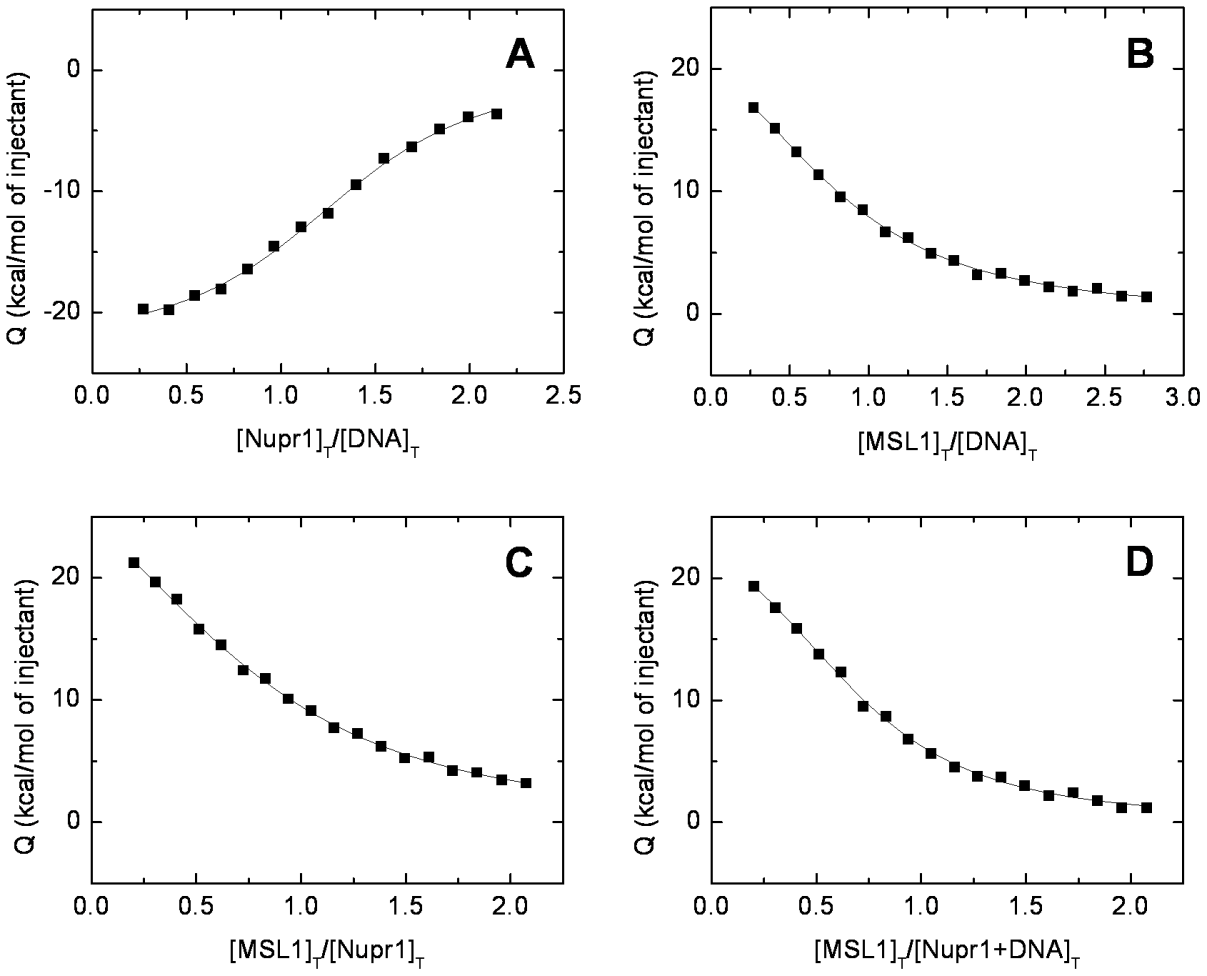
Calorimetric titrations for Nupr1, MSL1 and chemically-damaged-DNA interactions. Binding isotherms (normalized injection heat *versus* molar ratio of the reactants in the cell) are shown: (A) Nupr1 interacting with etoposide-damaged DNA. (B) MSL1 interacting with etoposide-damaged-DNA. (C) MSL1 interacting with Nupr1. (D) MSL1 interacting with Nupr1 + etoposide-damaged-DNA complex. All the titrations were carried out at 25°C in 10 mM acetate buffer (pH 4.5). The binding experiments involving damaged DNA were analyzed considering an estimated molecular weight of 10000 kDa.

Binding experiments involving etoposide-damaged-DNA were also carried by ITC (Fig. 6) and we could determine the apparent affinity of complexes (considering an estimated molecular weight of 10000 kDa for damaged DNA). Data obtained from these experiments showed a higher affinity of MSL1 for the etoposide-damaged-DNA + Nupr1 complex (*K*_D_ = 0.8±0.1 µM; Fig. 6 D) compared to those for etoposide-damaged-DNA (*K*_D_ = 1.2±0.2 µM; Fig. 6 B) or Nupr1 (*K*_D_ = 2.8±0.7; Fig. 6 C), separately. This decrease in the *K*_D_s could be due to the presence of the pre-existing complex between Nupr1 and damaged-DNA (*K*_D_ = 0.4±0.1 µM; Fig. 6 A). The affinity of Nupr1 for etoposide-damaged-DNA was slightly higher, although similar within the error, to those measured for the other complexes (Table 1); however, the binding reaction was enthalpically-driven (Table 1). This finding suggests that the binding mechanism of MSL1 to etoposide-damaged-DNA or to the etoposide-damaged-DNA + Nupr1 complex was different to that of the binding reaction to isolated Nupr1.

The 2D ^1^H-^15^N HSQC NMR experiment in the presence of etoposide-damaged-DNA and MSL1 with an ^15^N-labelled Nupr1 showed the disappearance of signals of residues Gly45 to Arg83 (Figure. S3 of File S1) and several others at the N terminus of Nupr1, suggesting that all these residues were involved in direct binding to DNA (that is, roughly 50% of all signals). This result is different to that observed for the Nupr1 + MSL1 complex, where only a few signals disappeared (see above). On the other hand, from the observed residues those showing the largest CSPs were: Ala3-Thr4, Ala8-Gln13,Gly17-Ser23, Asp26-Ser28. Many of these hydrophobic patches were also involved in the binding of Nupr1 to MSL1 (see above), but the observed CSP values for the ternary complex were greater than for the Nupr1 + MSL1 complex. These residues were not directly involved in DNA or MSL1 binding, but rather their environment was altered upon addition of DNA. A similar behaviour (residues that disappear and others with variations in their chemical shifts) has also been observed in the complexes formed between several IDPs [39].

We also tried to run 2% agarose gel to test for binding to damaged DNA. But the chemical-damage caused signal smearing in the corresponding gel lane, impairing any further analysis.

## Discussion

### Nupr1 and MSL1 Bound under DNA-damaging Conditions in vivo and Protected of Cell Death

We have previously described that Nupr1 binds to MSL1; furthermore, we have suggested that this complex could participate in the DNA-repair processes as judged from a clonogenic survival assay [24]. In the present work, we found that formation of the Nupr1 + MSL1 complex was dependent on DNA-damage since in untreated cells this complex was almost absent (Fig. 1). However, we found that localization of the complex into the nucleus was not on the DNA-damaged sites, as judged by the γ-H2AX staining, suggesting that the damaged-DNA was only the inductor of the complex in a dose-dependent manner, but not the recruiter itself. Moreover, the Nupr1 + MSL1 complex was very efficient to protect the cells against cell death induced by cisplatin-mediated DNA damage. This effect was also confirmed on other pancreatic cancer-derived cells (Panc-1) and using glucose starvation as DNA-damage induction.

The location of the binding site on damaged-DNA was further supported by the NMR experiments. Binding of Nupr1 to non-damaged-DNA resulted in disappearance of all protein signals in the ^15^N-HSQC spectrum (Figure S4 of File S1). However, binding of Nupr1 to etoposide-damaged-DNA led to the disappearance of some (but not all) signals (Figure S5 of File S1), suggesting that the molecular weight of the DNA-protein complexes was not very large. If Nupr1 was bound to the particular damaged-DNA sites, large protein-DNA complexes would have been formed, and signals in the HSQC would have disappeared. In addition, the different pattern of signal broadening of Nupr1, in the presence of MSL1, upon addition of etoposide-damaged-DNA (Figs. S2 and S3) suggests that when the three biomolecules are present, Nupr1 was not interacting with the whole amount of DNA. Nupr1 is known to regulate transcription of several genes involved in DNA repair [21] through an unknown mechanism, whereas MSL1 is involved in regulating histone acetylation [11], [15], which is presumed to participate in chromatin remodelling facilitating transcription. Our current hypothesis is that the Nupr1 + MSL1 complex is induced by DNA-damage, recruited on DNA to facilitate expression of genes involved in DNA repair, and that the whole activity is regulated by MSL1. That is, we speculate that the protein-protein complex regulates the expression of certain genes by recognizing specific undamaged DNA; in this way, we can explain that the formed Nupr1 + MSL1 complex only co-localizes minimally with damaged sites *in vivo*, when both proteins bind to damaged DNA *in vitro* (see below). However, it is important to note that, at this stage, and since we have only used a isoform of MSL1, we do not know exactly the role of other homologs of MSL1 (MSLv1 or NSL proteins) in regulating Nupr1. Finally, it is important to note that other DNA-repairing proteins co-localize with DNA damaged foci, but this is not the case for MSL1; we suggest that the DNA recognition ability in MSL1 is, in turn, modulated by the presence of Nupr1.

### MSL1 Showed Some Features of IDPs

One of the results of this work was the possibility of isolating intact, human isoform 1 of MSL1 in enough amount for biophysical studies. The protein with a TRX-tag was purified from inclusion bodies, following a dilution refolding protocol. So far, only the polypeptide patch corresponding to the coiled-coil region had been co-expressed with MOF [16]. The expression of MSL1, together with previous structural studies of proteins described in our groups [26], suggests that TRX-tag is an efficient system to express difficult, aggregation-prone proteins.

There were previous studies that suggested theoretically that MSL1 was disordered [16]. However, there was no experimental evidence of the disorder in the whole, intact protein. We have shown, indirectly, that intact, isolated MSL1 did not have a stable hydrogen-bond network: the lack of sigmoidal behaviour in the thermal denaturations (monitored by fluorescence or CD) suggests that, if any, MSL1 showed very limited residual structure.

We could not determine the size of isolated MSL1 through gel filtration, due to protein binding to the column. However, since the protein showed a non-constant heat dilution in the ITC experiments, we could conclude that MSL1 self-associates. Self-association of the isolated coiled-coil region of MSL1 has been shown experimentally in the X-ray structure of its complex with MOF [16]. Moreover, self-associated species have been described in other IDPs involved in cell-regulation and signalling [40]. It is important to note that the coiled-coil region of MSL1 appeared well-folded only when bound to MOF, but so far there was no evidence of its conformational properties in the isolated MSL1. Our results below suggest that even in the presence of Nupr1 or DNA, MSL1 was not well-folded, but it was oligomeric.

### “Fuzzy” Complex Formation between MSL1 and Nupr1

MSL1 bound to Nupr1 with the smallest affinity (*K*_D_ = 2.8 µM). This value was slightly larger (but within the same order of magnitude) than that obtained from SPR measurements: 0.9 µM [24]. The three-fold difference can be due to the dissimilar conditions (SPR and ITC) where the measurements were carried out. The positive enthalpy of the binding process was the largest measured in this work (Table 1) and positive (unfavourable), indicating that the process was entropically-driven. This binding yielded a “fuzzy” (disordered) complex [40], as suggested by several pieces of evidence. First, the lack of thermal-denaturation transitions observed by CD and fluorescence suggests the absence of rigid hydrogen-bonds. Second, the absence of significant changes in the far-UV CD spectra upon binding suggests that there was not a well-formed stable, rigid complex. And finally, the absence of changes in the chemical shifts of the observable signals in the ^15^N-HSQC spectrum of Nupr1 (Figs. S2 and S3) suggests that this protein remained unfolded. Therefore, we propose that the increase observed in entropy upon binding must come from: (i) a redistribution of the states that affect flexibility/mobility in the conformational population of both isolated IDPs; and, (ii) the desolvation of the binding interfaces of both proteins.

In conclusion, the Nupr1 + MSL1 was a “fuzzy” (disordered) complex [40].

### “Fuzzy” Complex Formation between MSL1 and Etoposide-damaged-DNA

It has been shown that MSL1 binds to double-strand γ-irradiation-induced damaged-DNA, and that Nupr1 negatively up-regulates this binding [24]. In this work, we showed that MSL1 bound to chemically-damaged-DNA, but it did not bind to non-damaged-DNA. Therefore, MSL1 might act as a DNA-repair protein. The binding was entropically-driven, with a dissociation constant of 1.2 µM. However, thermal denaturations did not show the presence of a stable structure in the protein upon complex formation. Therefore, the increase in entropy upon binding must come, as in the case of the complex between MSL1 and Nupr1, from: (i) a redistribution of the states that affect flexibility/mobility in the conformational population of isolated MSL1; and, (ii) the desolvation of the binding interfaces of both molecules [41]. We conclude that MSL1 formed a random “fuzzy” (disordered) complex with DNA, where MSL1 maintains a high flexibility.

There are examples of such highly-dynamic complexes between proteins ([40] and references therein) and between proteins and nucleic acids [42], [43]. The disordered regions in IDPs, which remain unstructured upon binding, can: (i) facilitate diffusion along nucleic acids (to recognize several nucleic acid sequences and allow movement across the nucleic acid strands); and, (ii) allow selectivity by forming different and specific interactions with nucleic acids. It is interesting to note that the way by which MSL1 recognizes DNA was different to that used by Nupr1. The affinity of Nupr1 for DNA was slightly larger, although similar within the error, but the DNA + Nupr1 recognition was enthalpically-driven. The differences cannot be attributed to the formation of a more rigid complex, since, as judged from fluorescence and far-UV CD thermal denaturations, and NMR results (Figure S5 of File S1), the Nupr1 + etoposide-damaged-DNA complex was also “fuzzy”. These results suggest that the narrowing of the conformational states of both proteins, or, alternatively, the desolvation of the binding surface of Nupr1 induced by DNA binding was different to that occurring when the sole protein target was the isolated MSL1; this finding could be probably due to the different size of both proteins. Large and negative values of Δ*S* have been observed in binding involving IDPs leading to “fuzzy” complexes ([39] and references therein).

MSL1 also bound to the preformed complex etoposide-damaged-DNA + Nupr1, with a similar affinity to that of the binding of isolated MSL1 to chemically-damaged-DNA, although the enthalpy of binding was different. These results suggest that the binding mechanisms of the two processes were also different, and that MSL1 did not recognize the damaged-DNA alone when it was bound to Nupr1 (in agreement with siRNA results, which show that the MSL1 + Nupr1 complex recognized DNA). Furthermore, MSL1 recognized isolated Nupr1, but the thermodynamic parameters of the MSL1-Nupr1 interaction were also different to those measured in the other complexes (Table 1). As it happens with the MSL1 + etoposide-damaged-DNA binary complex, the ternary MSL1 + etoposide-damaged-DNA + Nupr1 complex was “fuzzy”.

Taken together, we suggest that the increased mobility in the formed complexes facilitates probing different interactions to localize the proper binding-site configuration. Thus, either from the Nupr1 or MSL1 view, DNA binding narrows the native state ensemble of the protein, favouring some conformers. In fact, as it has been suggested [42], [43], the disordered region, which maintains its conformational heterogeneity upon binding to DNA, can help to fine-tune the flexibility of the “chosen” bound states.

### The Interacting Regions of Nupr1

The ^15^N-HSQC spectra provide us with information about the binding sites in Nupr1. Based on the disappearance of signals, the results suggest that there were different recognition sites to form the binary complex with MSL1 and the ternary one with MSL1 and chemically-damaged-DNA. Only the regions around Ser10, Asp22 and Asp36 were present in both complexes (Figs. S2 B and S3 B). Interestingly enough, some of those regions are close to at least one of both tyrosine residues in the protein: Tyr31 and Tyr37, which were involved in the binding to prothymosin α [44] (further, Tyr31 disappeared upon Nupr1 + MSL1 complex formation suggesting its involvement in the binding site). Theoretical predictions of the ordered regions in Nupr1 involve the regions Ala3-Thr9 and Ser32-Ty37 [45], which are some of the polypeptide patches affected upon binding. Finally, a study of the flexibility of Nupr1, based on the chemical shifts of the assigned heteronuclei [46] reported in this work, suggests that the less flexible region of the protein is Leu30-Leu33, which does not comprise any of the polypeptide patches involved in binding to the several biomolecules. Therefore, we conclude that Nupr1 uses different, highly flexible polypeptide regions to recognize different molecules, although there are some residues (probably a “binding-scaffold”) in common. We suggest that these common patches would be the ideal targets to disrupt most of the interactions of Nupr1 with its multiple partners.

### Conclusions and Biological Implications

We were able to obtain intact, isoform 1 of human MSL1. The protein seemed to be disordered, and it remained so when bound to Nupr1 and DNA. We were able to measure, for the first time, the affinity of MSL1 towards damaged-DNA and we described how this activity was fine-tuned by the presence of Nupr1 in cells. The formed complexes were “fuzzy” (disordered) in all cases, probably due to the necessity of transient contacts among the molecules during different cell-cycle time-points, favouring fast association and dissociation. Therefore, the different regulation pathways where Nupr1 intervenes must involve a stochastic view of the structures of the different complexes involved. The fuzziness of the complexes further suggests that specificity/affinity determinants of DNA binding (or protein-binding) cannot be deduced from a static structure. The entire population of complexes of Nupr1 exist as a multiplicity of states, modulating their conformational equilibria by binding to other biomolecules, and its physiological activity along the several regulation pathways where it is involved [22], [23].

## Supporting Information

File S1 **Supplementary Data are available at PLoS One online as one combined file (S1).** A table (Table S1) comprising the chemical shifts of the backbone nuclei of Nupr1 (25°C, pH 4.5). Five figures showing the sequence of MSL1 used in our studies (Figure S1 in File S1); the superimposed HSQC spectra of isolated Nupr1 and its complex with MSL1 (Figure S2 in File S1); the superimposed HSQC spectra of isolated Nupr1 and its complex with MSL1 and chemically damaged DNA (Figure S3 in File S1); the superimposed HSQC spectra of isolated Nupr1 with that of Nupr1 in complex non-damaged DNA (Figure S4 in File S1); and the superimposed HSQC spectra of isolated Nupr1 with that of Nupr1 with chemically-damaged DNA (Figure S5 in File S1).
(DOC)
