# Supplementary material for: Deciphering the Binding between Nupr1 and MSL1 and Their DNA-Repairing Activity
Source: PLoS One. 2013 Oct 30;8(10):e78101. doi: 10.1371/journal.pone.0078101 (PMC3813506; doi:10.1371/journal.pone.0078101)

**Combined Supplementary File S1 for the manuscript by Aguado-llera et al**

Table ST1. Chemical shifts for Nupr1

| RESIDUE | ^1^HN (ppm) | ^15^N(ppm) | ^13^CO(ppm) | ^13^Cα(ppm) | ^13^Cβ(ppm) |
| --- | --- | --- | --- | --- | --- |
| -3G | 8.54 | 110.93 | 173.73 | 45.21 | - |
| -4S | 8.13 | 115.03 | 174.51 | 58.08 | 64.11 |
| -5H | 8.53 | 120.25 | 175.74 | 55.20 | 29.12 |
| 1G | 8.47 | 110.94 | 173.74 | 45.21 | - |
| 2S | 8.23 | 115.78 | 174.31 | 58.27 | 64.05 |
| 3A | 8.33 | 125.87 | 177.42 | 52.49 | 19.20 |
| 4T | 7.91 | 113.19 | 173.75 | 61.71 | 70.14 |
| 5F | 8.08 | 122.88 | 173.33 | 55.35 | 38.93 |
| 6P | - | 138.10 | 174.59 | 61.43 | 30.67 |
| 7P | - | 135.12 | 176.79 | 62.97 | 31.87 |
| 8A | 8.39 | 123.96 | 178.15 | 52.62 | 19.07 |
| 9T | 8.00 | 112.35 | 174.47 | 61.72 | 69.85 |
| 10S | 8.14 | 117.30 | 173.54 | 57.91 | 63.91 |
| 11A | 8.12 | 126.75 | 175.28 | 50.61 | 18.17 |
| 12P | - | 135.59 | 176.90 | 63.17 | 31.81 |
| 13Q | 8.38 | 120.26 | 175.70 | 55.80 | 29.52 |
| 14Q | 8.26 | 122.44 | 173.41 | 53.47 | 28.81 |
| 15P | - | 138.60 | 174.63 | 61.43 | 30.12 |
| 16P | - | 135.33 | 177.10 | 63.15 | 32.00 |
| 17G | 8.30 | 109.38 | 177.11 | 44.34 | - |
| 18P | - | 134.14 | 177.45 | 63.54 | 32.11 |
| 19E | 8.59 | 119.94 | 176.21 | 56.33 | 29.57 |
| 20D | 8.12 | 120.57 | 176.28 | 54.51 | 41.19 |
| 21E | 8.21 | 120.77 | 176.24 | 56.58 | 30.09 |
| 22D | 8.33 | 121.01 | 176.44 | 54.43 | 41.08 |
| 23S | 8.20 | 116.74 | 174.83 | 58.78 | 64.06 |
| 24S | 8.30 | 117.75 | 174.56 | 58.87 | 63.87 |
| 25L | 8.01 | 123.22 | 177.12 | 55.12 | 42.18 |
| 26D | 8.24 | 120.87 | 176.35 | 54.22 | 41.16 |
| 27E | 8.33 | 121.29 | 176.68 | 57.13 | 29.57 |
| 28S | 8.27 | 115.86 | 174.89 | 59.47 | 63.89 |
| 29D | 8.22 | 122.06 | 176.69 | 54.81 | 40.94 |
| 30L | 7.94 | 121.29 | 178.01 | 56.36 | 41.89 |
| 31Y | 7.98 | 118.90 | 176.82 | 58.87 | 38.11 |
| 32S | 7.96 | 116.03 | 175.44 | 59.44 | 63.64 |
| 33L | 8.05 | 123.12 | 177.84 | 56.21 | 41.99 |
| 34A | 7.90 | 121.80 | 177.89 | 52.89 | 18.80 |
| 35H | 7.98 | 115.73 | 174.50 | 55.43 | 28.50 |
| 36S | 7.99 | 115.84 | 174.44 | 58.65 | 63.91 |
| 37Y | 8.12 | 121.95 | 175.96 | 57.92 | 38.15 |
| 38L | 8.05 | 123.60 | 177.67 | 55.25 | 41.99 |
| 39G | 7.84 | 108.59 | 174.71 | 45.51 | - |
| 40G | 8.18 | 108.51 | 174.81 | 45.43 | - |
| 41G | 8.31 | 108.71 | 174.83 | 45.45 | - |
| 42G | 8.24 | 108.48 | 174.26 | 45.09 | - |
| 43R | 8.13 | 120.38 | 176.40 | 56.22 | 30.59 |
| 44K | 8.32 | 122.20 | 176.87 | 56.46 | 32.79 |
| 45G | 8.31 | 109.82 | 173.94 | 45.06 | - |
| 46R | 8.17 | 120.49 | 176.49 | 56.15 | 30.79 |
| 47T | 8.18 | 115.61 | 174.47 | 62.02 | 70.19 |
| 48K | 8.34 | 123.92 | 176.57 | 56.72 | 32.95 |
| 49R | 8.32 | 122.75 | 176.52 | 56.41 | 30.38 |
| 50E | 8.36 | 122.48 | 176.18 | 56.45 | 30.27 |
| 51A | 8.27 | 125.16 | 177.49 | 52.41 | 19.10 |
| 52A | 8.15 | 123.12 | 177.53 | 52.41 | 18.92 |
| 53A | 8.14 | 122.91 | 177.61 | 52.53 | 19.25 |
| 54N | 8.30 | 117.67 | 175.46 | 53.18 | 38.75 |
| 55T | 8.00 | 113.71 | 174.31 | 62.01 | 69.87 |
| 56N | 8.34 | 120.88 | 174.63 | 53.21 | 38.78 |
| 57R | 8.13 | 122.32 | 173.91 | 53.96 | 30.02 |
| 58P | - | 137.03 | 176.70 | 62.88 | 31.95 |
| 59S | 8.45 | 117.77 | 173.01 | 56.44 | 63.36 |
| 60P | - | 138.27 | 177.54 | 63.57 | 31.64 |
| 61G | 8.38 | 109.11 | 174.57 | 45.14 | - |
| 62G | 8.20 | 108.40 | 174.35 | 45.23 | - |
| 63H | 8.36 | 117.94 | 174.56 | 55.14 | 29.03 |
| 64E | 8.52 | 121.93 | 176.30 | 56.67 | 29.94 |
| 65R | 8.37 | 122.85 | 176.18 | 56.29 | 30.32 |
| 66K | 8.30 | 123.25 | 176.15 | 56.22 | 32.98 |
| 67L | 8.24 | 124.40 | 177.05 | 55.00 | 42.36 |
| 68V | 8.15 | 122.30 | 176.29 | 62.17 | 32.68 |
| 69T | 8.16 | 118.97 | 174.10 | 61.99 | 69.88 |
| 70K | 8.26 | 124.04 | 176.31 | 56.39 | 33.22 |
| 71L | 8.21 | 123.73 | 177.36 | 55.23 | 42.34 |
| 72Q | 8.37 | 121.40 | 176.00 | 56.00 | 29.42 |
| 73N | 8.45 | 120.14 | 175.73 | 53.82 | 38.90 |
| 74S | 8.27 | 116.26 | 174.79 | 58.95 | 63.94 |
| 75E | 8.31 | 122.70 | 176.60 | 56.96 | 30.05 |
| 76R | 8.19 | 121.87 | 176.66 | 56.51 | 30.38 |
| 77K | 8.15 | 121.84 | 176.58 | 56.50 | 32.91 |
| 78K | 8.20 | 122.51 | 176.67 | 56.33 | 32.80 |
| 79R | 8.32 | 122.59 | 176.63 | 56.34 | 30.44 |
| 80G | 8.37 | 110.36 | 173.61 | 45.27 | - |
| 81A | 8.08 | 123.66 | 177.48 | 52.56 | 19.56 |
| 82R | 8.27 | 120.64 | 175.25 | 56.13 | 30.49 |
| 83R | 7.91 | 127.40 | 180.79 | 57.43 | 31.43 |

Figure S1: Sequence of the 413-residue-long MSL1 used in our studies (Genbank accession number CR749360; Swiss-Prot: Q68DK7)

MRKSPLGGGGGSGASSQAACLKQILLLQLDLIEQQQQQLQAKEKEIEELKSERDTLLAR

IERMERRMQLVKKDNEKERHKLFQGYETEEREETELSEKIKLECQPELSETSQTLPPKP

FSCGRSGKGHKRKSPFGSTERKTPVKKLAPEFSKVKTKTPKHSPIKEEPCGSLSETVCK

RELRSQETPEKPRSSVDTPPRLSTPQKGPSTHPKEKAFSSEIEDLPYLPTTEMYLCRWH

QPPPSPLPLRESSPKKEETVARCLMPSSVAGETSVLAVPSWRDHSVEPLRDPNPSDLLE

NLDDSVFSKRHAKLELDEKRRKRWDIQRIREQRILQRLQLRMYKKKGIQESEPEVTSFF

PEPDDVESLMITPFLPVVAFGRPLPKLTPQNFELPWLDERSRCRLEIQKKQTPHRTCRK

Figure S2 (A): Overlay of ^1^H-^15^N HSQC spectra of native Nupr1 (black) and Nupr1 with MSL1 (red). Experiments were acquired in a 800 MHz spectrometer.


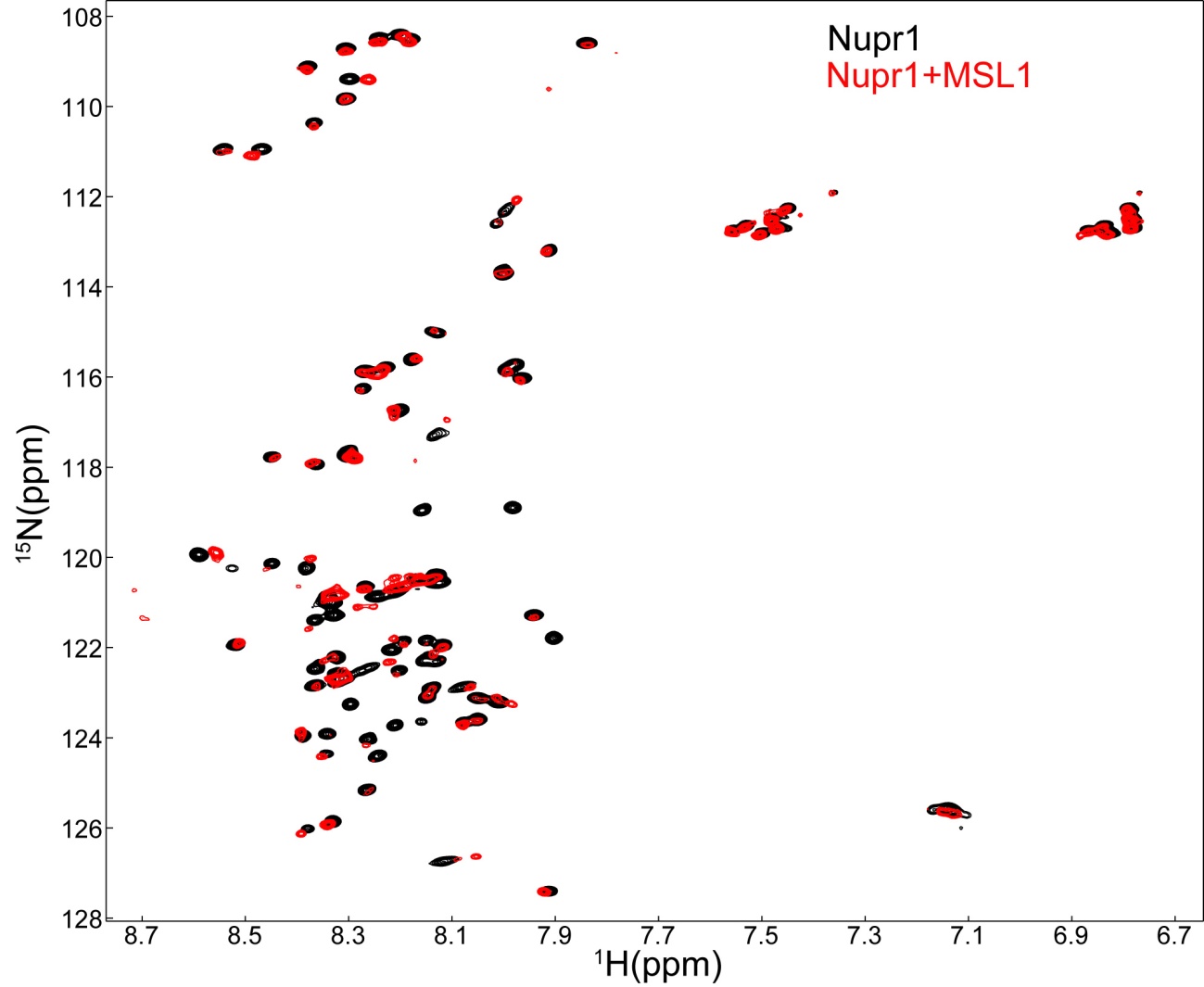


Figure S2 (B): Chemical Shift Perturbation after addition of MSL1 to Nupr1. Proline residues are indicated by asterisks.


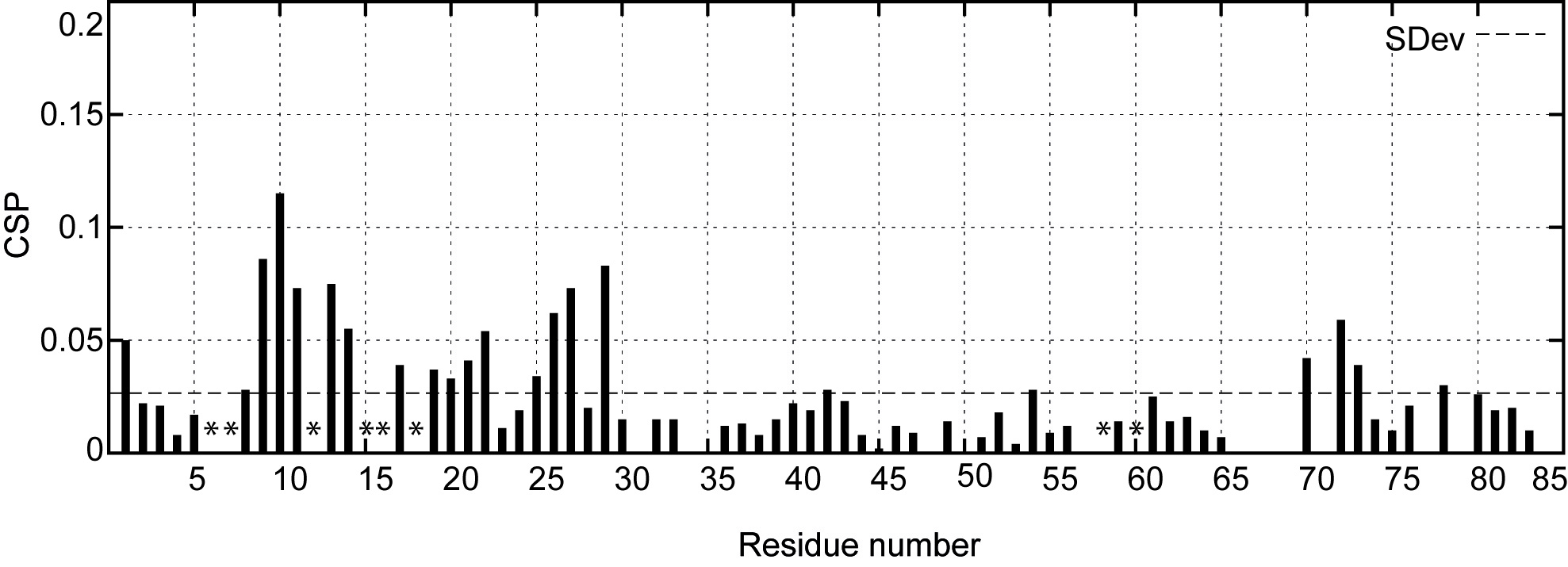


Figure S3 (A): Overlay of ^1^H-^15^N HSQC spectra of native Nupr1 (black) and Nupr1 with MSL1 and etoposide-damaged DNA (red). Experiments were acquired in a 800 MHz spectrometer.


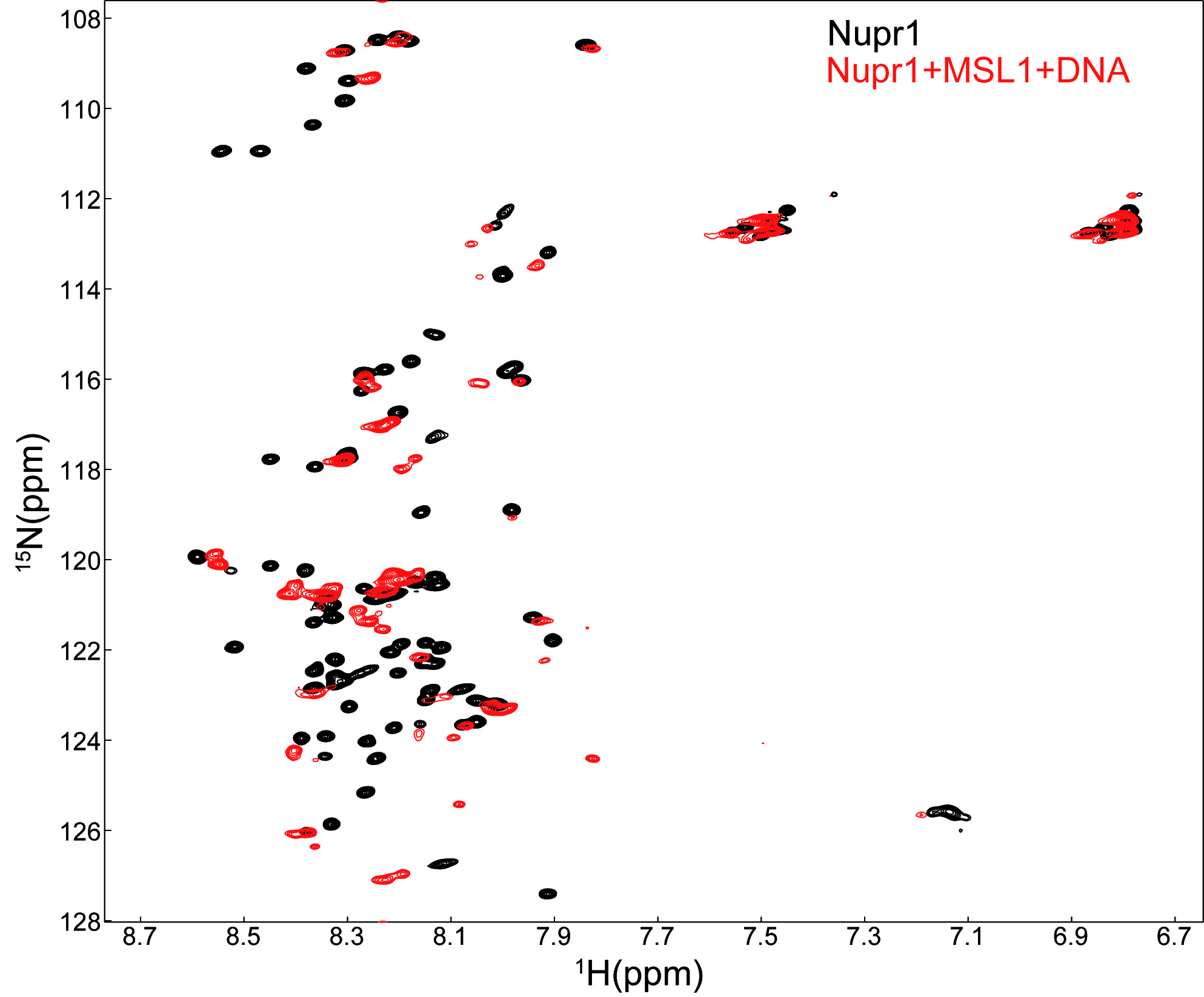


Figure S3 (B): Chemical Shift Perturbation after addition of MSL1 and etoposide-damaged-DNA to Nupr1. Proline residues are indicated by asterisks.


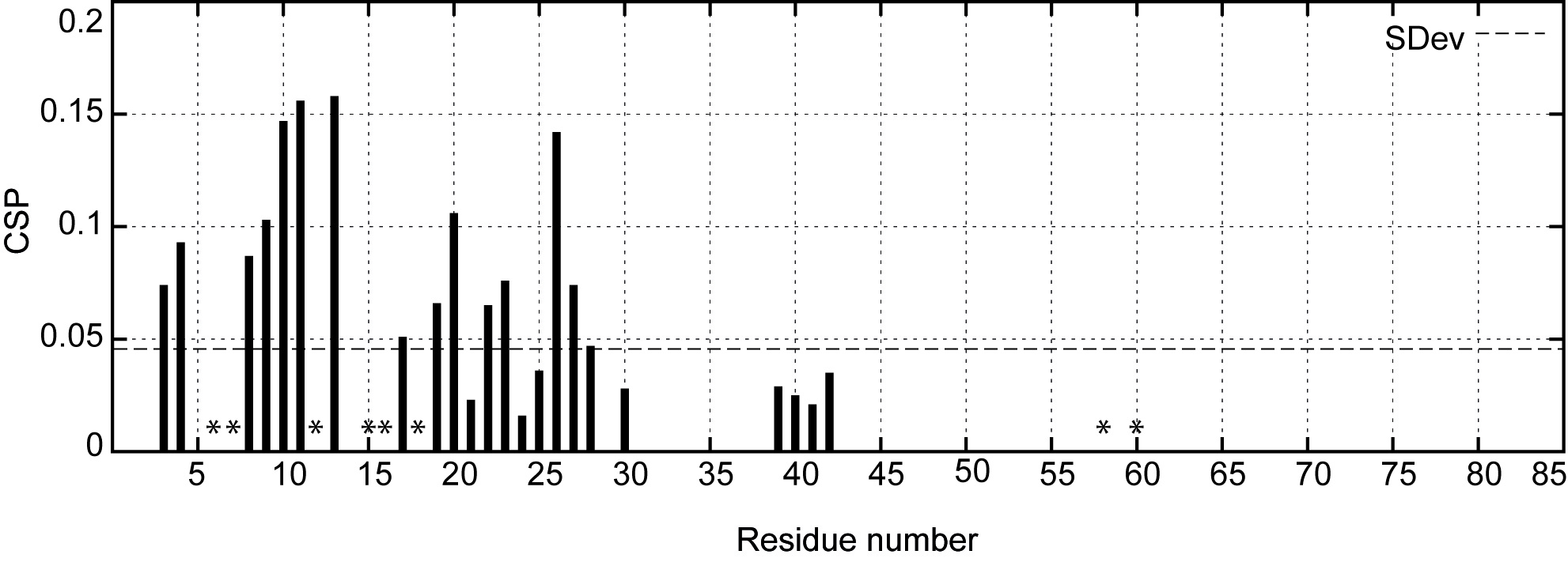


Figure S4: Overlay of ^1^H-^15^N HSQC spectra of native Nupr1 (black) and Nupr1 with non-damaged DNA (red). Experiments were acquired in a 500 MHz spectrometer.


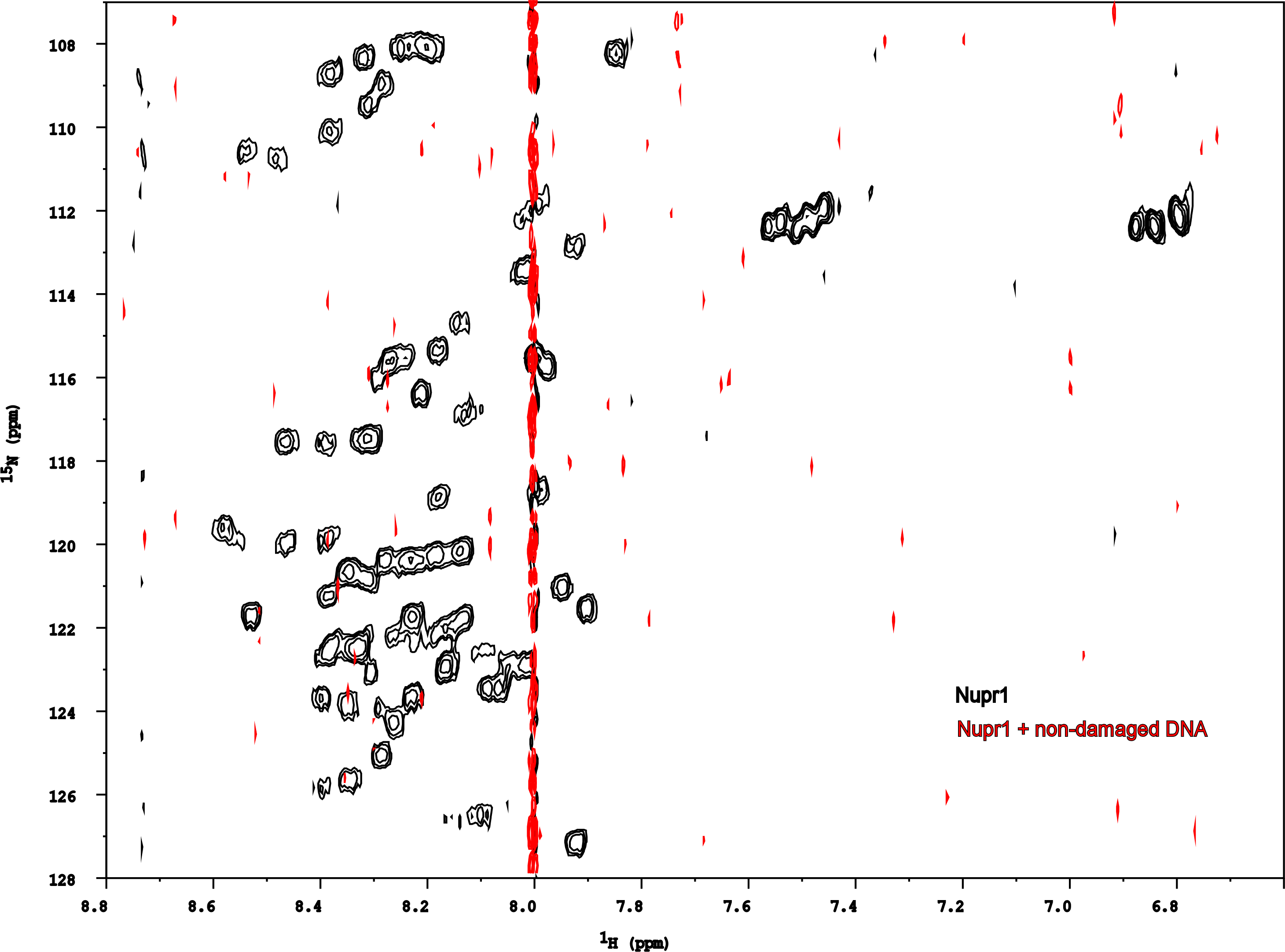


Figure S5: Overlay of ^1^H-^15^N HSQC spectra of native Nupr1 (black) and Nupr1 with etoposide-damaged DNA (red). Experiments were acquired in a 500 MHz spectrometer.


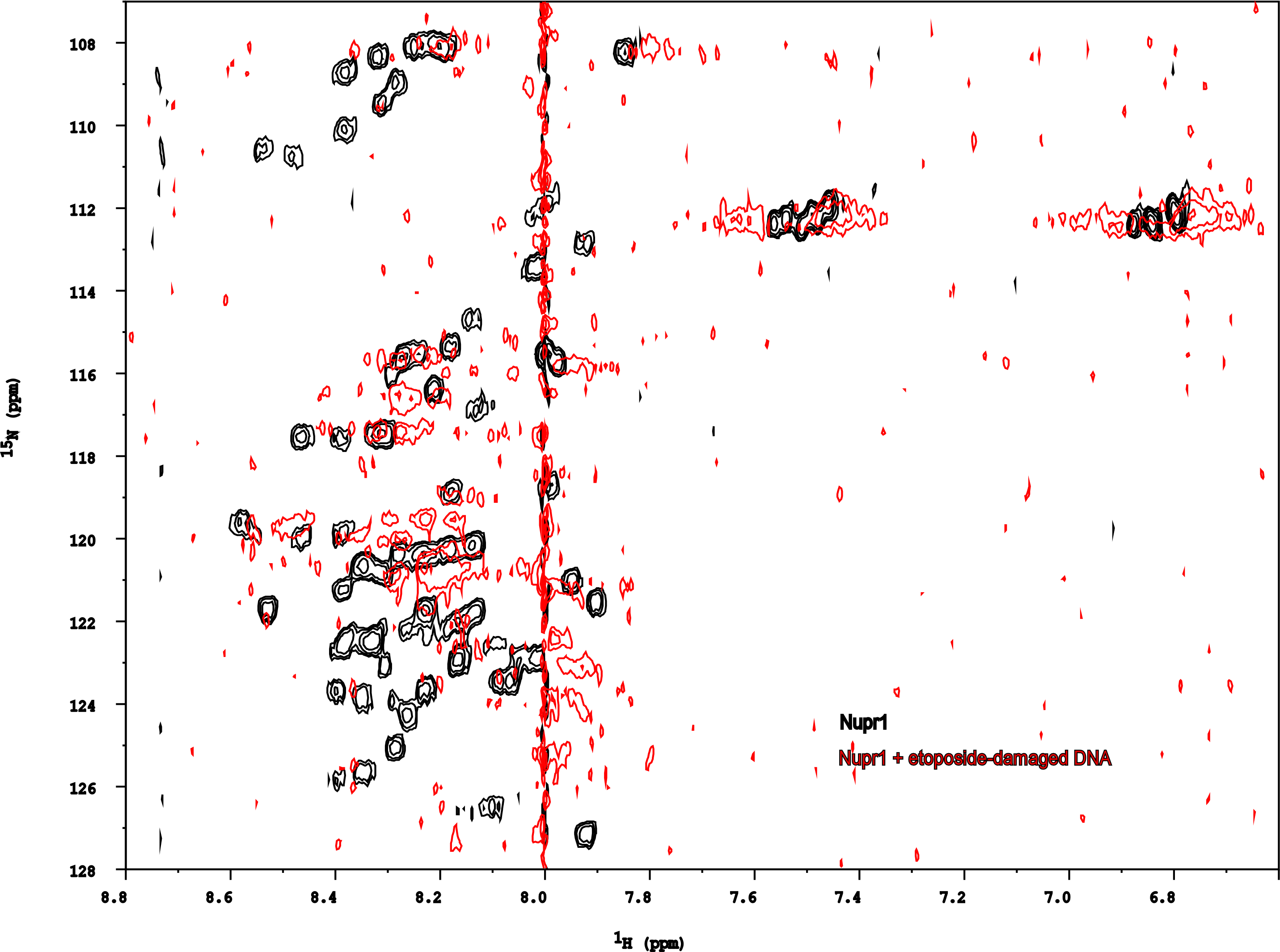

Supplement: File S1 — Supplementary Data are available at PLoS One online as one combined file (S1). A table (Table S1) comprising the chemical shifts of the backbone nuclei of Nupr1 (25°C, pH 4.5). Five figures showing the sequence of MSL1 used in our studies (Figure S1 in File S1); the superimposed HSQC spectra of isolated Nupr1 and its complex with MSL1 (Figure S2 in File S1); the superimposed HSQC spectra of isolated Nupr1 and its complex with MSL1 and chemically damaged DNA (Figure S3 in File S1); the superimposed HSQC spectra of isolated Nupr1 with that of Nupr1 in complex non-damaged DNA (Figure S4 in File S1); and the superimposed HSQC spectra of isolated Nupr1 with that of Nupr1 with chemically-damaged DNA (Figure S5 in File S1). (DOC) [file pone.0078101.s001.doc]
